# Identification of hepatic protein-protein interaction targets for betaine homocysteine S-methyltransferase

**DOI:** 10.1371/journal.pone.0199472

**Published:** 2018-06-20

**Authors:** Francisco Garrido, María Pacheco, Rocío Vargas-Martínez, Roberto Velasco-García, Inmaculada Jorge, Horacio Serrano, Francisco Portillo, Jesús Vázquez, María Ángeles Pajares

**Affiliations:** 1 Instituto de Investigaciones Biomédicas Alberto Sols (CSIC-UAM), Arturo Duperier 4, Madrid, Spain; 2 Cardiovascular Proteomics Group, Spanish National Center for Cardiovascular Research (CNIC) and CIBERCV, Melchor Fernández de Almagro 3, Madrid, Spain; 3 Department of Internal Medicine, University of Puerto Rico, Medical Sciences Campus, San Juan, Puerto Rico; 4 Instituto de Investigación Sanitaria La Paz (IdiPAZ), Paseo de la Castellana 261, Madrid, Spain; 5 Departamento de Bioquímica, Facultad de Medicina, Universidad Autónoma de Madrid, Arzobispo Morcillo 4, Madrid, Spain; 6 Centro de Investigación Biomédica en Red de Cáncer (CIBERONC), Instituto de Salud Carlos III, Madrid, Spain; 7 Departamento de Biología Estructural y Química, Centro de Investigaciones Biológicas (CSIC), Ramiro de Maeztu 9, Madrid, Spain; University of Illinois, UNITED STATES

## Abstract

Protein-protein interactions are an important mechanism for the regulation of enzyme function allowing metabolite channeling, crosstalk between pathways or the introduction of post-translational modifications. Therefore, a number of high-throughput studies have been carried out to shed light on the protein networks established under different pathophysiological settings. Surprisingly, this type of information is quite limited for enzymes of intermediary metabolism such as betaine homocysteine S-methyltransferase, despite its high hepatic abundancy and its role in homocysteine metabolism. Here, we have taken advantage of two approaches, affinity purification combined with mass spectrometry and yeast two-hybrid, to further uncover the array of interactions of betaine homocysteine S-methyltransferase in normal liver of *Rattus norvegicus*. A total of 131 non-redundant putative interaction targets were identified, out of which 20 were selected for further validation by coimmunoprecipitation. Interaction targets validated by two different methods include: S-methylmethionine homocysteine methyltransferase or betaine homocysteine methyltransferase 2, methionine adenosyltransferases α1 and α2, cAMP-dependent protein kinase catalytic subunit alpha, 4-hydroxyphenylpyruvic acid dioxygenase and aldolase b. Network analysis identified 122 nodes and 165 edges, as well as a limited number of KEGG pathways that comprise: the biosynthesis of amino acids, cysteine and methionine metabolism, the spliceosome and metabolic pathways. These results further expand the connections within the hepatic methionine cycle and suggest putative cross-talks with additional metabolic pathways that deserve additional research.

## Introduction

High levels of homocysteine (Hcy) in plasma have been associated with a variety of pathologies that expand from cardiovascular disease to hearing loss [1–4]. The liver, and precisely, impairments in hepatic methionine metabolism seem to be responsible of these increased plasmatic Hcy concentrations, since approximately 50% of the ingested methionine is used in this organ [5]. Hcy is produced in the methionine cycle, catabolized by the transsulfuration pathway and its excess is exported to the blood [6]. However, when there is a need of methionine, Hcy can be methylated for the synthesis of this amino acid. Three enzymes can perform Hcy methylation using different methyl donors and, among them betaine homocysteine S-methyltransferase (BHMT) uses the osmolyte betaine for this purpose [7]. The diet is the main source of betaine [8], but this metabolite can be also obtained by choline oxidation in the mitochondria [9]. This oxidation allows recovery of one out of the three methyl groups donated by S-adenosylmethionine for the synthesis of phosphatidylcholine from phosphatidylethanolamine in one of the hepatic processes that more S-adenosylmethionine consumes [10]. Therefore, BHMT becomes a link between osmoregulation, phospholipid synthesis and methionine/Hcy metabolism.

BHMT is mainly expressed in the liver [11, 12], where it constitutes ~1% of the total protein of hepatocytes [7, 13, 14]. Nevertheless, different levels of *Bhmt* gene expression are also detected in other rat tissues [11, 15, 16]. Intermediate expression levels are measured in the kidney, followed by the testis and brain, and low levels are found in the lung, cerebellum and skeletal muscle [11]. Moreover, in tissues with low expression levels the BHMT protein is only detected in specific cell types (i.e. Sertoli and Purkinje cells) [11]. *BHMT* gene expression is altered in several diseases mainly concerning the liver (i.e. cirrhosis), but also in diabetes [17] or Barret’s esophagus [18]. Moreover, the presence of the protein in the lens has been associated to its role in osmoregulation, a process that is altered during cataract development [19].

BHMTs are highly conserved and sequences for the human and rat proteins are ~92% identical. The enzyme is a homotetramer with high thermal stability [20, 21], a property that has been used for its purification from mammalian tissues. Crystal structures show the tight binding between BHMT monomers in the dimer, as well as that of the dimers in the tetramer [22, 23]. An essential contributor to the high enzyme stability is the existence of a C-terminal α-helix (~30 residues) that extends from one monomer towards another located immediately below or above, establishing a large number of hydrophobic interactions [20, 21, 24]. The rest of the subunit folds into a (α/β)_8_ barrel, where Zn^2+^ binding takes place through three cysteines and a tyrosine [22, 23, 25].

Classical studies have considered BHMT a cytoplasmic enzyme, but recent data show BHMT immunostaining both in the cytoplasm and the nuclear compartment in most cell types. Nevertheless, the cytoplasmic localization is preferred in hepatocytes, whereas nuclear localization is favored in tissues/cells exhibiting low expression [11]. Importantly, BHMT has the same oligomeric assembly in both subcellular compartments, where it is able to catalyze methionine synthesis [11]. In normal liver, only minute amounts of nuclear BHMT can be detected, but reductions in the cytosolic protein content together with nuclear accumulation are detected upon induction of acute liver injury with D-galactosamine or inhibition of glutathione synthesis by buthionine sulfoximine [11]. This anomalous nucleocytoplasmic distribution can be prevented by the administration of N-acetylcysteine or glutathione monoethyl ester, two compounds that serve as glutathione precursors and aid to the preservation of the normal GSH/GSSG ratio.

Mechanisms that have been involved in the control of nucleocytoplasmic distribution include the addition or removal of posttranslational modifications (PTMs), as well as protein-protein interactions. Datasets derived from several high-throughput studies focused on the identification of protein targets for certain PTMs or on the elucidation of the human interactome have rendered data concerning BHMT. For example, phosphorylation [26, 27], ubiquitination [28], acetylation [29, 30], succinylation [30], carbonylation [31], and nitrosylation [32] are some of the PTMs detected on BHMT in these analyses, whereas other studies also identify BHMT as a target for modification by transglutaminase [33]. Nevertheless, in many cases the existence of these PTMs has not been verified by additional methods, neither the enzymes catalyzing these modifications have been identified. Additionally, datasets from high-throughput experiments also showed the involvement of BHMT in some protein-protein interactions, which constitutes a surprisingly small set given the hepatic protein abundance. This list includes e.g. BHMT2 and high mobility group box 1 (HMGB1), which needs to be translocated to the cytoplasm for its interaction with BHMT [34–36]. Therefore, in order to expand the current network of BHMT hepatic interacting partners we have carried out a new high-throughput study using a double approach, affinity purification combined with mass spectrometry (AP-MS) and yeast two-hybrid (YTH). Here, we describe the new targets identified, as well as their validation by coimmunoprecipitation.

## Materials and methods

### Plasmid constructions and mutagenesis

The pTYB12-BHMT and pFLAG-BHMT plasmids containing the ORF of *R*. *norvegicus Bhmt* were described previously [11, 25]. Plasmids containing the sequences of BHMT interaction targets were prepared from Image clones containing the complete ORFs that were purchased from Source Bioscience Geneservice (Nottingham, UK). Exceptions are: i) pHA-MAT containing the rat *Mat1a* ORF that was previously described [37]; ii) pHA-MAT2A that was obtained by NdeI/EcoRI digestion of pT7.7-MAT2A [38], cloning of the ORF into a modified pBluescript containing a NdeI restriction site, EcoRI digestion of pBS(NdeI)-MAT2A and ligation of the ORF in the same site of pCMV-HA (Clontech, Mountain View, CA, USA); and iii) pHA-ALDOB and pHA-HPD that were prepared from total *R*. *norvegicus* liver RNA. The ORFs were amplified using the Superscript^TM^ one-step RT-PCR kit (Invitrogen, Carlsbad, CA, USA), the primers and RT-PCR conditions described in Table 1. Cloning into pCMV-HA (Clontech) was carried out to obtain the corresponding HA-tagged proteins, whose linker sequences are listed in Table 2. The molecular weight of the tagged proteins was calculated using the ExPASy Compute pI/Mw tool (http://web.expasy.org/compute_pi/).

**Table 1:** ORFs of BHMT interaction targets, PCR programs and primers used for amplification.

Protein code or name	Source or IMAGE clone	origin	Amplification primers^a^	Restriction sites	PCR program
A7VJC2	2822109	human	5’-cggaattcgaaatcgggctgaagcgact-3’	**EcoRI KpnI**	95°C 2 min (95°C 30s; 56° 1 min; 72°C 90s) x 30 72°C 10 min
			5’-ggggtaccgtgaagcccatggcaaatag-3’		
Q9ESW0	3845478	human	5’-ggggtaccttcgcttgtgtccctctttct-3’	**KpnI NotI**	95°C 2 min (95°C 30s; 56° 1 min; 72°C 210s) x 30 72°C 10 min
			5’-atagtttagcggccgcctttggggagggtcagca-3’		
O88902^b^	6579163	human	5’-cggaattcggcgattcggcacgag-3’	**EcoRI XhoI**	95°C 2 min (95°C 30s; 56°C 60s; 72°C 3 min) x 30 72°C 10 min
			5’-cgtgcgctcgagcag-3’		
			5’- ccgctcgagcgcacgcagt-3’	**XhoI XhoI**	95°C 2 min (95°C 30s; 56°C 60s; 72°C 3 min) x 30 72°C 10min
			5’-tcgctcgagaggaccaggtaggcaaaacc-3’		
P60711	6920838	rat	5’-cggaattcacaaccttcttgcagctcctc-3’	**EcoRI KpnI**	95°C 2 min (95°C 30s; 56° 1 min; 72°C 90s) x 30 72°C 10 min
			5’-ggggtaccaagggtgtaaaacgcagctc-3’		
Q6DUV1	40028213	human	5’-ggggtaccccgaccatggtagtgttcaa-3’	**KpnI NotI**	95°C 2 min (95°C 30s; 55° 1 min; 72°C 150s) x 30 72°C 10 min
			5’-ataatttagcggccgccatcggcaaagtccaactg-3’		
Q9JLS3	9020643	human	5’-cgagatctgccaggccccactctcagg-3’	**BglII NotI**	95°C 2 min (95°C 30s; 56° 1 min; 72°C 4 min) x 30 72°C 10 min
			5’-ataatttagcggccgcctggaagggctggagtcag-3’		
Q56R17	4834709	human	5’-ggggtaccacgggggaaggagtcacc-3’	**KpnI NotI**	95°C 2 min (95°C 30s; 58° 1 min; 72°C 90s) x 30 72°C 10 min
			5’-atagtttagcggccgcgctgcattgtacctaacttcca-3’		
P23514	5599357	rat	5’-ggggtaccgccatgaccgcagctgagaa-3’	**KpnI NotI**	95°C 2 min (95°C 30s; 56° 1 min; 72°C 210s) x 30 72°C 10 min
			5’-atagtttagcggccgcacactttcgaggaccgtttg-3’		
P63018	7104230	rat	5’-cggaattcaagcctacacgcaagcaacc-3’	**EcoRI KpnI**	95°C 2 min (95°C 30s; 56° 1 min; 72°C 120s) x 30 72°C 10 min
			5’-ggggtaccgaacaatgctataccctctactttga-3’		
P62961	7105037	rat	5’-ggggtaccacagtcaccatcaccgcaac-3’	**KpnI NotI**	95°C 2 min (95°C 30s; 54° 1 min; 72°C 1 min) x 30 72°C 10 min
			5’-atagtttagcggccgcgaccaaaccggatgatggta-3’		
P13383	7109097	rat	5’-cggaattcccgccatcatggtgaaa-3’	**EcoRI NotI**	95°C 2 min (95°C 30s; 55° 1 min; 72°C 150s) x 30 72°C 10 min
			5’-ataatttagcggccgcctattcaaacttcgtcttctttcc-3’		
P14659	7112083	rat	5’-cggaattcattggtcactccgaccagtca-3’	**EcoRI KpnI**	95°C 2 min (95°C 30s; 56° 1 min; 72°C 120s) x 30 72°C 10 min
			5’-ggggtaccaggtttacgcggactccag-3’		
B1WC97	7114289	rat	5’-cggaattctactgcttgactttagttcttcagg-3’	**EcoRI KpnI**	95°C 2 min (95°C 30s; 56° 1 min; 72°C 1 min) x 30 72°C 10 min
			5’-ggggtaccgattcttgtttcccaggacaat-3’		
B4F7A9	7114331	rat	5’-ggggtaccacagtttcggtccggattc-3’	**KpnI NotI**	95°C 2 min (95°C 30s; 56° 1 min; 72°C 90s) x 30 72°C 10 min
			5’-atagtttagcggccgcgaggaaccgcaacagacc-3’		
P63159	7128547	rat	5’-cggaattctcgcggaggaaaatcaactaa-3’	**EcoRI KpnI**	95°C 2 min (95°C 30s; 56° 1 min; 72°C 1 min) x 30 72°C 10 min
			5’-ggggtaccgggggttaaatgctttatagacaa-3’		
Q5XIQ3	7134100	rat	5’-cggaattcaagcagtttgcaggctctcc-3’	**EcoRI KpnI**	95°C 2 min (95°C 30s; 56° 1 min; 72°C 1 min) x 30 72°C 10 min
			5’-ggggtaccgagggcagctttgggttc-3’		
P27791	7314651	rat	5’-ggggtacccagccgcgtcgcagctc-3’	**KpnI NotI**	95°C 2 min (95°C 30s; 62° 1 min; 72°C 90s) x 30 72°C 10 min
			5’-atagtttagcggccgcagaaaacccatggggcaca-3’		
P04256	7374689	rat	5’-cggaattcaacgctctcatcatcctaccg-3’	**EcoRI KpnI**	95°C 2 min (95°C 30s; 56° 1 min; 72°C 1 min) x 30 72°C 10 min
			5’-ggggtaccaagctttgtttcctggctgt-3’		
Q4V8C1	7456915	rat	5’-ggggtaccggacttctcaccccaaacct-3’	**KpnI NotI**	95°C 2 min (95°C 30s; 58° 1 min; 72°C 90s) x 30 72°C 10 min
			5’-ataatttagcggccgcgcaagttgagaccttgcac-3’		
ALDOB	Liver RNA	rat	5’-cgaattccgatggctcaccgatttccag-3’	**EcoRI XhoI**	50°C 30 min 94°C 2 min (94°C 15s; 58°C 30s; 70°C 75s) x 36 72°C 10 min
			5’-ccgctcgagggtgacggtatctagtagg-3’		
HPD	Liver RNA	rat	5’-cgaattccgatgacaacctacagcaac-3’	**EcoRI XhoI**	50°C 30 min 94°C 2 min (94°C 15s; 40°C 30s; 70°C 75s) x 36 72°C 10 min
			5’-ccgctcgagttacattccagacctcac-3’		

^a^restriction sites appear underlined

^b^amplification of two fragments containing XhoI restriction sites was done independently and the whole ORF obtained upon ligation

**Table 2:** Linkers of the HA-tagged proteins used in this study.

Protein code or name	No linker residues	Amino acid sequence of the linker	Tagged protein size (kDa)^a^
A7VJC2	18	LMAMEARIRNRAEATESA	40.69
Q9ESW0	38	LMAMEARIRSTEISRGTFACVPLSSVALESRRAAPSLD	42.77
O88902	21	LMAMEARIRRFGTRGRRVPAA	167.07
P60711	26	LMAMEARIHNLLAAPPSPVHTRHQFA	45.84
Q6DUV1	19	LMAMEARIRSTEISRGTPT	86.89
Q9JLS3	25	LMAMEARIRSTEICQAPLSGPPGAT	142.55
Q56R17	26	LMAMEARIRSTEISRGTTGEGVTGPA	61.81
P23514	18	LMAMEARIRSTEISRGTA	110.20
P63018	15	LMAMEARIQAYTQAT	73.77
P62961	24	LMAMEARIRSTEISRGTTVTITAT	39.54
P13383	11	LMAMEARIPAI	79.56
P14659	16	LMAMEARIHWSLRPVR	72.80
B1WC97	34	LMAMEARILLLDFSSSGLRPPAYPKPRPPPRARG	38.05
B4F7A9	31	LMAMEARIRSTEISRGTTVSVRIPGIPVPPA	45.70
P63159	16	LMAMEARILAEENQLN	27.94
Q5XIQ3	25	LMAMEARIQAVCRLSQQRGLGLGPT	36.52
P27791	33	LMAMEARIRSTEISRGTQPRRSSGTGPGRDAAA	45.30
P04256	16	LMAMEARIQRSHHPTV	37.29
Q4V8C1	26	LMAMEARIRSTEISRGTGLLTPNLPL	46.95
MATα1	9	LMAMEAEFH	45.86
MATα2	13	LMAMEARIPLWHH	46.46
HPD	9	LMAMEARIP	47.34
ALDOB	9	LMAMEARIP	41.75

^a^Calculated using ExPASy tools

Plasmids for YTH were prepared from pBS-BHMT that contains the ORF of rat *Bhmt* [25]. For this purpose, pBS-BHMT was NdeI/BamHI digested and the ORF cloned into the same sites on pGBKT7 (Clontech) to render pGBKT7-BHMT. Cloning into pACT2 (Clontech) required site-directed mutagenesis of pBS-BHMT to create a NcoI restriction site using the QuikChange method and the primers 5’-GATATCGAATTCCATGGCACCGATTGCC-3’ (sense; NcoI site underlined) and its complementary. The mutated plasmid was NcoI/BamHI digested and cloned into pACT2 to obtain pACT2-BHMT. Sequences were verified by automatic sequencing at the Genomic Service of the Instituto de Investigaciones Biomédicas “Alberto Sols” (IIBM).

### Production of intein and intein-BHMT

Competent *E*. *coli* BL21(DE3) cells were transformed with either pTYB12 or pTYB12-BHMT and grown in LB plates containing 50 μg/ml ampicillin (LBA). A single colony was used to inoculate 100 ml of LBA medium that was grown overnight at 37°C. This culture was used to inoculate 3 liters of LBA medium that were further incubated at 37°C to reach A_600_~0.9. At this point, expression of the proteins was induced by addition of 0.5 mM IPTG overnight at 22°C. Cells were harvested by centrifugation and the pellet was divided in aliquots (1 g) for storage at -80°C.

Bacteria overexpressing intein (6 g) or intein-BHMT (2 g) were lysed on ice in 20 mM Tris/HCl pH 8, 500 mM NaCl, 0.1 mM EDTA, 0.1% Triton X-100 (buffer A) containing protease inhibitors (2 μg/ml aprotinin, 1 μg/ml pepstatin A, 2.5 μg/ml antipain, 0.5 μg/ml leupeptin, 0.1 mM PMSF, 0.1 mM benzamidine) in a Branson 250 sonifier (30 cycles on/off 30s; out power level 8). Lysates were cleared by centrifugation at 13000 xg for 30 min at 4°C, the supernatants collected and used to load chitin columns (New England Biolabs, Ipswich, MA, USA).

### Preparation of liver cytosolic samples

Male *R*. *norvegicus* Wistar (~250g; N = 10) received standard diets *ad libitum* and were euthanized by CO_2_ asphyxiation. The livers were immediately extracted, washed with PBS and frozen using liquid nitrogen for storage at -80°C. Cytosolic fractions were prepared immediately before use by homogenization of liver samples (10 g) in 20 ml of 10 mM sodium phosphate buffer pH 7.4, 5 mM EDTA, 0.1 mM EGTA (buffer B) containing protease inhibitors (2 μg/ml aprotinin, 1 μg/ml pepstatin A, 2.5 μg/ml antipain, 0.5 μg/ml leupeptin, 0.1 mM PMSF, 5 mM benzamidine and 10 μg/ml soybean trypsin inhibitor). Homogenates were centrifuged at 15000 xg 20 min at 4°C and the supernatant centrifuged for 1h at 105000 xg and 4°C to obtain the cytosols. The experiments included in this study were approved by the CSIC Bioethics Committee and carried out in full accordance with Spanish regulations (RD 53/2013) and the European Community guidelines (2010/63/EU) for the use of laboratory animals.

### Preparation of columns and isolation of protein interaction targets

Three columns containing chitin beads (New England Biolabs) were prepared for each of the nine independent experiments carried out. Column 1 (4 ml) was equilibrated with buffer B, whereas columns 2 (4 ml) and 3 (1 ml) were equilibrated in buffer A and loaded with intein and intein-BHMT lysates, respectively. After binding of the baits, columns 2 and 3 were also equilibrated in buffer B before loading of rat liver protein samples. All columns were run at 10 ml/h and 4°C.

Column 1 was loaded with liver cytosol and the flowthrough collected and loaded into column 2. The flowthrough of this second column was also collected and loaded into column 3. The three columns were then extensively washed with buffer B until A_280_ ~0. At this point elution of the proteins bound was carried out with a gradient from 0–500 mM NaCl (40 column volumes). Fractions (5 ml for columns 1 and 2; 1 ml for column 3) were collected during all the process and A_280_ recorded. Columns 2 and 3 were later incubated with 3 column volumes of buffer B containing 30 mM 2-mercaptoethanol for 48 h at 23°C for intein excision, allowing recovery of strong interactors together with BHMT. Samples (50 μl) of the loaded cytosols and flowthroughs, as well as from the eluted peaks were separated on 10% SDS-PAGE gels, which were later stained with Coomassie Blue. Protein peaks were washed extensively with 20 mM ammonium acetate pH 7.7 using PM-10 membranes (AMICON Inc. Beverly, MA, USA). The concentrated protein pools were then lyophilized, reconstituted with water (50 μl) and divided in aliquots to measure protein concentration and to prepare for SDS-PAGE (10–30 μg/lane) by addition of Laemmli buffer.

### Mass spectrometry detection of protein interaction targets

Proteins were in-gel digested using a previously described protocol [39]. Briefly, identical amounts of each protein sample were suspended in sample buffer and loaded in 2.8-cm-wide wells of an SDS-PAGE gel. The run was stopped as the front entered 3 mm into the resolving gel. The protein band was visualized by Coomassie Blue staining, excised, and digested overnight at 37°C with 60 ng/l trypsin (Promega, Madison, WI, USA; Cat. No. V5111) at a 5:1 (w/w) protein:trypsin ratio in 50 mM ammonium bicarbonate pH 8.8, containing 10% (v/v) acetonitrile and 0.01% (w/v) 5-cyclohexyl-1-pentyl-D-maltoside (Fluka; Cat. No. 96193). The supernatant was recovered and the gel pieces incubated with 12 mM ammonium bicarbonate pH 8.8 for 1 hour. Both supernatants were combined and 25% (v/v) trifluoroacetic acid (Merck, Darmstadt, Germany; Cat. No. 808260) added until pH 3. Peptides were finally desalted using OMIX tips (Varian Inc., Walnut Creek, CA, USA; Cat. No. A57003100) following manufacturer’s instructions and dried down. Isolated peptides were analyzed by LC-MS/MS using a Surveyor LC system coupled to a linear ion trap mass LTQ spectrometer (Thermo Fisher) as previously described [40]. The LTQ was operated in a data-dependent MS/MS mode using the 15 most intense precursors detected in a survey scan from 400 to 1,600 m/z [40, 41]. Number of microscans, normalized collision energy, and dynamic exclusion parameters were as previously described [40, 41]. Protein identification was carried out as described previously using the SEQUEST algorithm (Bioworks 3.2 package, Thermo Finnigan) [40, 42]. The MS/MS raw files were searched against the Rat Uniprot database (UniProt release 06/2009) supplemented with porcine trypsin and human keratin. The same collections of MS/MS spectra were also searched against inverted databases constructed from the same target databases. SEQUEST results were analyzed using the probability ratio method [43], and false discovery rates (FDR) were calculated using the refined method [44]. Peptide and scan counting was performed assuming as positive events those with an FDR lower than 5%.

### Yeast two-hybrid

Screening for BHMT interactions was carried out with a rat liver Matchmaker cDNA library (RL4004AH; Clontech) and the AH109 yeast strain. Transformation was performed with EasyComp solutions (Invitrogen) and selection achieved using low (-Leu/-Trp; LW) and high stringency (-Ade/-His/-Leu/-Trp; -AHLW) SC media for growth of the resulting transformants. Screening of 1.5 x 10^7^ clones was carried out and 52 putative interactions detected, from which only 38 were confirmed in–AHLW SC medium. DNAs of the positive clones were isolated and used to transform *E*. *coli* DH5α competent cells. Plasmids were purified with Qiagen plasmid purification kits (Qiagen, Hilden, Germany) and sequenced at the IIBM Genomic Service. Fourteen biologically relevant preys were found, the rest corresponding to BHMT-BHMT interactions; this large background was expected from the homo-oligomeric association state of BHMT and guarantees native folding of the fusion protein used for screening. Verification of positive interactions was carried out by cotransformation of plasmids harboring the *Bhmt* ORF and putative preys and growth on–AHLW SC media.

### Transient transfections and immunoprecipitation procedures

Commercial COS-7 (monkey kidney), N2a (mouse neuroblastoma) or H35 (rat hepatoma) cell lines were obtained from the IIBM collection and originally purchased from the ATCC. Cells were grown in DMEM (Gibco, Carlsbad, CA, USA) supplemented with 10% (v/v) fetal bovine serum and 2 mM glutamine. Cells (2x10^6^) were transfected using lipofectamine (Invitrogen) for 6 hours using pFLAG-BHMT, pHA-prey plasmids or cotransfected with pFLAG-BHMT/pHA-prey plasmids (10 μg) at 1:1 ratios. Total lysates were prepared 48 hours after transfection in 200 μl of 50 mM Tris/HCl pH 7.5, 150 mM NaCl, 1 mM EDTA, 1% (v/v) NP-40, 1 mM DTT containing 1 mM PMSF, 1 mM benzamidine, 2 μg/ml aprotinin, 1 μg/ml pepstatin A, 0.5 μg/ml leupeptin, 2.5 μg/ml antipain (immunoprecipitation buffer) by incubation on ice for 10 min, followed by six passages through a 25G syringe. Input samples were taken at this step, whereas the remaining sample was centrifuged for 15 min at 10000 xg and 4°C.

Cellular fractions (150 μl total lysates or 100 μl cytosolic fractions) were incubated o/n at 4ºC with mouse monoclonal anti-FLAG M2 Agarose (50 μl; Sigma, St. Louis, MI, USA; Cat. No. A2220) for anti-FLAG immunoprecipitation. On the other hand, the cellular fractions were precleared using anti-mouse IgG (2 μg) for 2 hours at 4°C before anti-HA immunoprecipitation as previously described [45]. Samples were then centrifuged for 15 min at 10000 xg before overnight incubation with mouse monoclonal anti-HA (2 μg, Covance; Cat. No. MMS-101R) coupled to protein A Sepharose CL-4B (GE Healthcare, Uppsala, Sweden) at 4°C. The beads were washed 3 times with immunoprecipitation buffer and later boiled in Laemmli sample buffer (40 μl) containing 100 mM DTT for 10 min. Following centrifugation for 5 min at 10000 xg, the supernatants were loaded on SDS-PAGE gels and proteins were electrotransferred to nitrocellulose membranes for immunoblotting.

### Immunoblotting

Membranes were incubated with mouse monoclonal anti-FLAG M2 (5 μg/ml, Sigma; Cat. No. F3165), mouse monoclonal anti-HA (1:1000 v/v, Covance; Cat. No. MMS-101R) or rabbit polyclonal anti-BHMT (1:20000 v/v) [25] as required. To avoid hindrance with mouse immunoglobulin bands after anti-FLAG or anti-HA immunoprecipitation, mouse TrueBlot ULTRA (1:1000 v/v, eBioscience; Cat. No. 18–8817) was used. Proteins were visualized using Western Lightning™ chemiluminescence reagent (Perkin Elmer, Waltham, MA, USA). The images were scanned and quantification carried out using ImageJ software.

### Determination of protein concentration

The protein concentration of the samples was measured using the Bio-Rad protein assay kit and bovine serum albumin as the standard.

### Protein-protein interaction network and statistical analysis

The list of BHMT-protein interactors obtained by AP-MS and YTH was analyzed using STRING (https://string-db.org/) and Bioprofiling (http://www.bioprofiling.de) tools for the identification of protein-protein interaction networks [46–48]. Student’s t-test for unpaired samples was applied for statistical analysis using GraphPad Prism v. 5.0 (GraphPad Software, San Diego, CA, USA). Data are shown as the mean ± SEM and differences were considered significant when p≤0.05.

## Results

**Fig 1:**
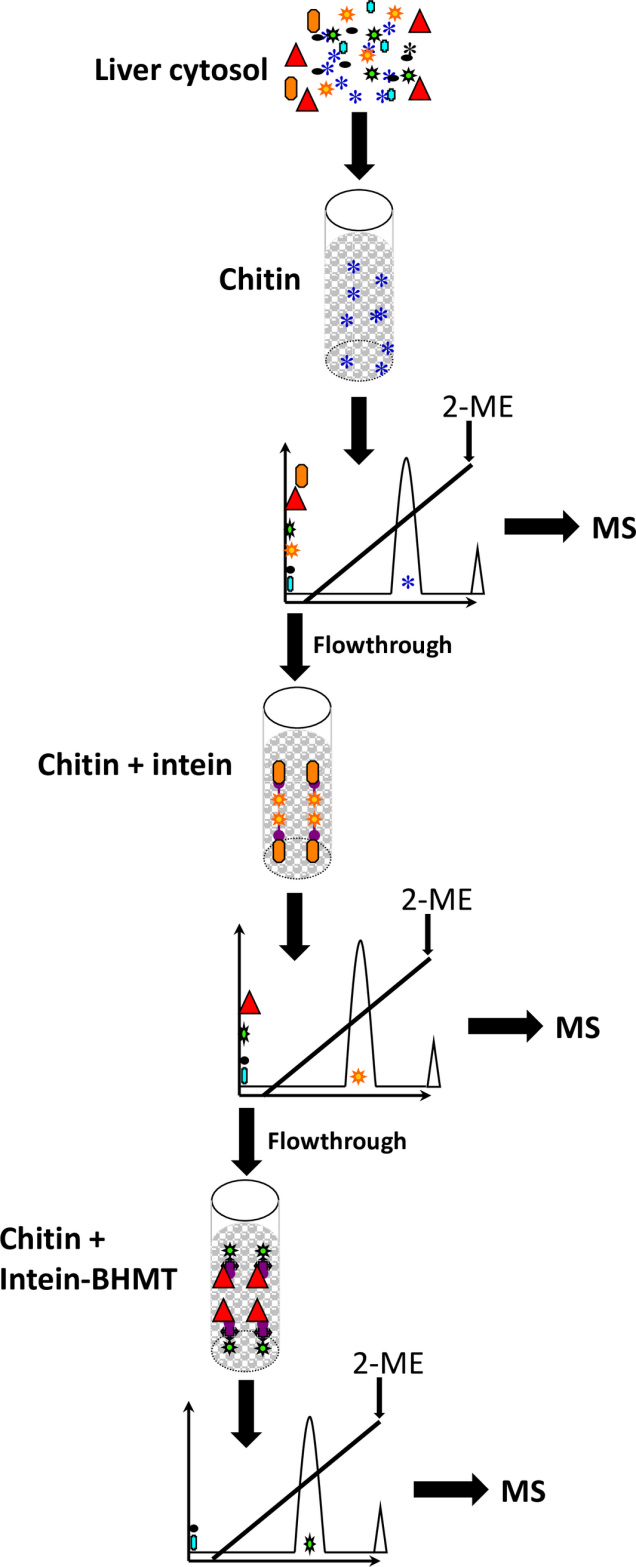
Schematic representation of the affinity purification/mass spectrometry procedure. Three chitin columns were used: column 1 contained only the beads (4 ml); column 2 contained intein bound to the beads (4 ml); and column 3 has intein-BHMT bound to the beads (1 ml). Liver cytosol was loaded on column 1, the flowthrough recovered and loaded on column 2 and the flowthrough of this last column loaded on column 3. Elution of the proteins bound to each of the columns was first performed with a salt gradient and finally, by incubation with 2-mercaptoethanol. A_280_ was measured during all the procedure to identify the eluted protein peaks that were collected and digested with trypsin for mass spectrometry identification of the interaction targets.

**Fig 2:**
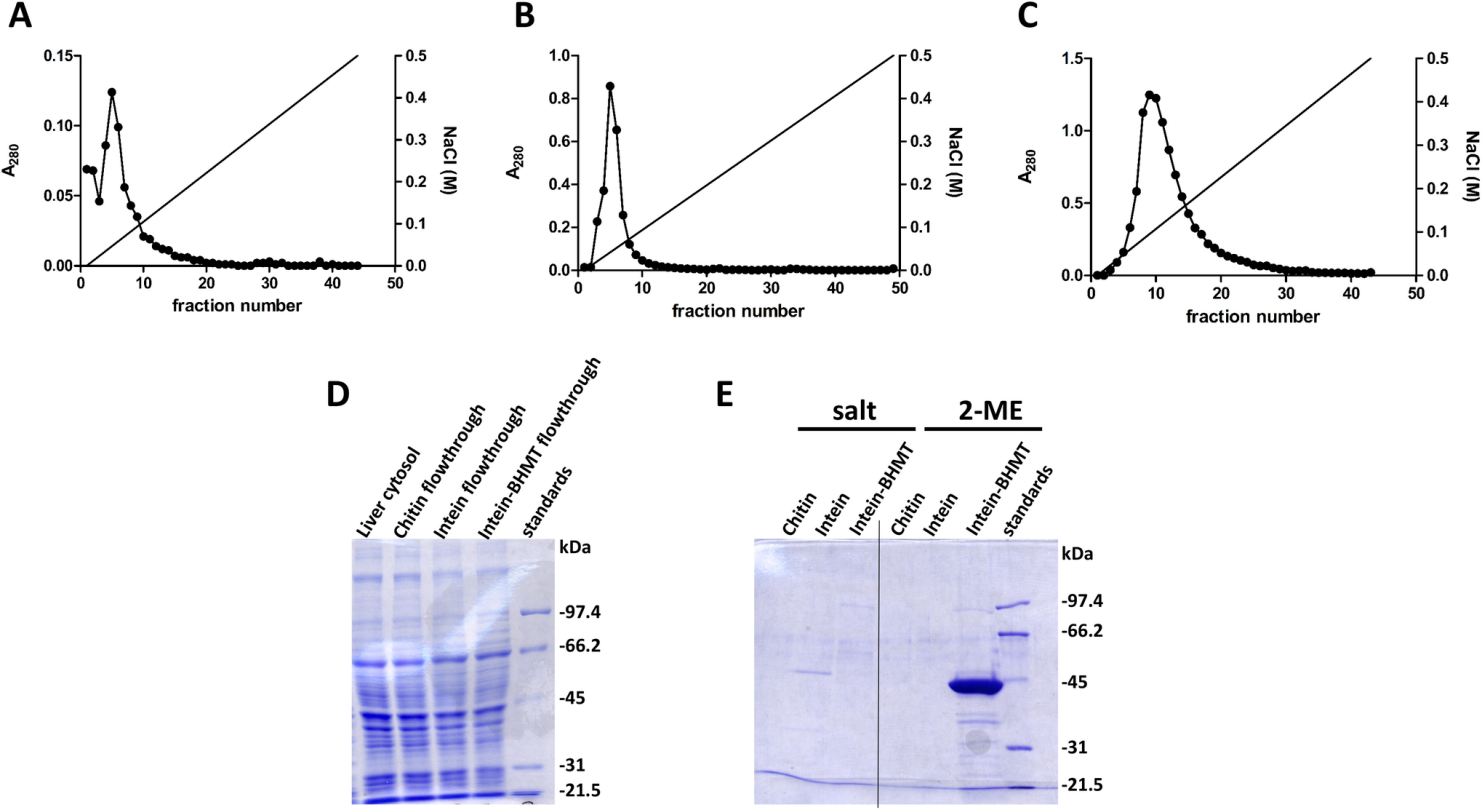
Elution profiles from control, intein and intein-BHMT loaded chitin columns. The figure shows representative A_280_ elution profiles from control (beads only; 4 ml), intein (4 ml) and intein-BHMT (1 ml) loaded chitin columns, as well as representative Coomassie Blue stained SDS-PAGE gels of liver cytosol, flowthroughs loaded on these columns and samples of the eluted peaks. (A) Control columns were loaded with liver cytosol and eluted with a NaCl gradient. (B) The flowthrough from the control column was loaded onto the intein column and elution performed with the same salt gradient. (C) The flowthrough of the intein column was loaded onto the intein-BHMT column and elution carried out with a NaCl gradient. (D) SDS-PAGE of the protein fractions loaded onto the columns: liver cytosol (2 μl); flowthrough of the chitin column (2 μl); flowthrough of the intein column (2 μl); and flowthrough of the intein BHMT column (2 μl). (E) Eluted proteins using salt gradients and 2-mercaptoethanol (2-ME) excision were collected and analyzed by SDS-PAGE: chitin peak (40 μl); intein peak (40 μl); and intein-BHMT peak (40 μl). Molecular weight standards are shown on the right side of each gel.

The search for BHMT interaction targets was performed initially by AP-MS using liver cytosols of Wistar rats. Elimination of unspecific interactors was carried out by sequential passages through control (beads only) and intein columns, before loading onto intein-BHMT columns (Fig 1). Proteins bound to each column were then eluted using a salt gradient and the A_280_ registered. For the three columns, the absorption profiles showed a single peak between 50–100 mM NaCl that was collected (Fig 2). Elution of stronger interactors required excision of the intein-BHMT tag by incubation with a reducing agent. In this case, BHMT (45 kDa) was recovered together with its strong interacting partners as observed in the corresponding gels (Fig 2E); again, the pools were collected. Identification of the proteins bound in each case was carried out after trypsinization by mass spectrometry. First, comparison between datasets corresponding to control and intein columns versus datasets of the intein-BHMT column was performed for proteins eluting with the salt gradient. Sixty-nine potential BHMT interaction targets were identified, which showed the same binding behavior in independent experiments (Table 3 and S1 Table). The same analysis was carried out for stronger interactors eluting upon excision of the intein tag and 59 putative interaction targets were identified in several experiments (Table 4 and S1 Table). Nevertheless, some targets of potential interest that were found only in one experiment were considered also for further validation. Among them, proteins of the methionine cycle, MATα1, MATα2 and BHMT2.

**Table 3:** Candidates for BHMT interaction identified by mass spectrometry after affinity purification on chitin columns eluted with a salt gradient.

Uniprot code	Protein
**P13444**^**a**^	**S-adenosylmethionine synthase isoform type-1, MATα1**
**P18298**	**S-adenosylmethionine synthase isoform type-2, MATα2**
Q5U2R0	Methionine adenosyltransferase 2 subunit beta, MAT**β**^b^
Q6P688	S-adenosylmethionine synthase, MAT**α**2^b^
Q63432	RAT Rat protein kinase C-family related^b^
**Q6DUV1**	**RAT Protein kinase C epsilon type**^b^
**B4F7A9**	**Casein kinase 2 α2**
Q9JIH7	Serine/threonine-protein kinase WNK1
**O88902**	**Tyrosine-protein phosphatase non-receptor type 23, PTPN23**
**Q5XIQ3**	**CXXC-type zinc finger protein 5, CXXC5**
**P60711**	**ACTB Actin, cytoplasmic 1 (Beta-actin).**
**Q4V8C1**	**RGD1306462 protein**
**P14659**	**HSP72 Heat shock-related 70 kDa protein 2**
**P63018**	**HSP7C Heat shock cognate 71 kDa protein**
**Q56R17**	**Importin subunit alpha, Karyopherin alpha 4**^b^
**B1WC97**	**BTB/POZ domain-containing protein KCTD7**
**P23514**	**Coatomer subunit beta**
**A7VJC2**	**Heterogeneous nuclear ribonucleoproteins A2/B1**
**P04256**	**Heterogeneous nuclear ribonucleoprotein A1**
**P63159**	**High mobility group protein B1 (HMGB-1)**
**P13383**	**Nucleolin**
**P62961**	**Nuclease-sensitive element-binding protein 1**
**Q9ESW0**	**DNA damage-binding protein 1**
P52925	High mobility group protein 2 (HMGB-2)
P17132	Heterogeneous nuclear ribonucleoprotein D0
Q6URK4	Heterogeneous nuclear ribonucleoprotein A3
Q8VHV7	Heterogeneous nuclear ribonucleoprotein H
Q66HM7	Sjogren syndrome antigen B, SSB
P13084	Nucleophosmin
P52590	Nuclear pore complex protein Nup107^b^
P43138	DNA-(apurinic or apyrimidinic site) lyase, APEX1
Q63396	Activated RNA polymerase II transcriptional coactivator p15
Q6AY09	Heterogeneous nuclear ribonucleoprotein H2
B0BN99	High mobility group box 3, Hmgb3
B0BNC9	Quinone oxidoreductase-like protein 2
O35796	C1QBP Complement component 1 Q subcomponent-binding protein, mitochondrial
Q6P6R6	Transglutaminase 2, C polypeptide^b^
Q9WVJ6	Tissue-type transglutaminase^b^
P17764	Acetyl-CoA acetyltransferase, mitochondrial
P28042	SSBP1 Single-stranded DNA-binding protein, mitochondrial
B0BND0	ENPP6 Ectonucleotide pyrophosphatase/phosphodiesterase family member 6
Q672K1	NADPH oxidase 3^b^
P35281	RAB10 Ras-related protein Rab-10^b^
Q7TPK6	Serine/threonine-protein kinase WNK4^b^
Q9Z286	Adenylate cyclase type 10^b^
Q6IMF3	Keratin, type II cytoskeletal 1
Q5EAP4	Guanine nucleotide binding protein, alpha 14
P04785	Protein disulfide-isomerase^b^
P06761	78 kDa glucose-regulated protein^b^
P13107	Cytochrome P450 2B3^b^
Q6LCX1	Cytochrome P450 2B3^b^
P07896	ECHP Peroxisomal bifunctional enzyme
Q9Z2M4	DECR2 Peroxisomal 2,4-dienoyl-CoA reductase
Q6AYD3	Proliferation-associated protein 2G4
B2RYX0	Naca protein
A1A5S1	Pre-mRNA splicing factor 6
Q6WAY2	Phospholipid phosphatase-related protein type 1
P47245	Nardilysin
Q6VV72	Eukaryotic translation initiation factor 1A
B1H281	LOC499754 protein
B5DFF5	Triobp protein
O88311	AlF-C1
B0LT89	Serine/threonine-protein kinase 24^b^
P12749	60S ribosomal protein L26
P62864	40S ribosomal protein S30 (Fau)
Q07205	Eukaryotic translation initiation factor 5
B2RZB7	Small nuclear ribonucleoprotein D1
B5DEN5	Eukaryotic translation elongation factor 1 beta 2
Q9QYQ9	Homeobox protein

^a^In bold, proteins selected for validation by immunoprecipitation.

^b^Proteins that are found in only one experiment, but are related to BHMT protein function, subcellular localization or previously described as BHMT protein-protein interaction targets.

**Table 4:** Candidates for BHMT interaction identified by mass spectrometry after affinity purification on chitin columns eluted with 2-mercaptoethanol.

Uniprot code	Protein
**Q9JLS3**^**a**^	**Serine/threonine-protein kinase TAO2**
**P27791**^**a**^	**cAMP-dependent protein kinase catalytic subunit alpha**^b^
**O88902**^**a**^	**Tyrosine-protein phosphatase non-receptor type 23**
P51577	P2X purinoceptor 4
Q62969	Prostacyclin synthase
A1A5S1	Pre-mRNA splicing factor 6
Q6WAY2	Phospholipid phosphatase-related protein type 1
Q5BK24	Uncharacterized protein C8orf76 homolog
Q3T1I3	Usher syndrome type-1C protein-binding protein 1
B2GV26	LOC304239 protein
Q5BJT0	Arginine and glutamate-rich protein 1^b^
Q5XIC3	Hsp90 co-chaperone Cdc37-like 1^b^
A2IA98	40S ribosomal protein S3^b^
O08875	Serine/threonine-protein kinase DCLK1^b^
P52796	Ephrin-B1^b^
P97531	Cdc42-interacting protein 4^b^
Q00939	Forkhead box protein G1^b^
Q5SGD7	Connector enhancer of kinase suppressor of ras 3^b^
B2GUW9	RGD1562161 protein^b^
B5DFC1	Vps35 protein^b^
Q4V8M7	LOC304239 protein^b^
Q5BJR5	Microspherule protein 1^b^
Q5U2M4	DNA ligase^b^
Q6P7B6	Ephrin B1^b^
Q8CH93	Hyaluronan synthase 1^b^
Q8QZV1	High affinity cGMP-specific 3’,5’-cyclic phosphodiesterase 9A^b^
Q9QYU6	Zinc finger protein^b^
Q80W87	Roundabout homolog 4^b^
A0JPQ3	Microtubule associated scaffold protein 2^b^
O35762	Homeobox protein Nkx-6.1^b^
Q07936	Annexin A2^b^
Q5M821	Protein phosphatase 1H^b^
Q5PQK1	Septin-10^b^
Q6AXN8	Zinc finger and SCAN domain containing 21^b^
Q8VHU4	Elongator complex protein 1^b^
Q923J6	Dynein heavy chain 12, axonemal^b^
Q9EQH1	GRB2-associated-binding protein 2^b^
O70199	UDP-glucose 6-dehydrogenase^b^
P05539	Collagen alpha-1(II) chain^b^
P23457	3-alpha-hydroxysteroid dehydrogenase^b^
P32821	Trypsin V-A^b^
P60669	Pleckstrin homology domain-containing family A member 4^b^
P70673	ATP-sensitive inward rectifier potassium channel 11^b^
Q09167	Serine/arginine-rich splicing factor 5^b^
Q4V8G7	Centromere protein U^b^
Q5M7W4	Transmembrane channel-like protein 5^b^
Q5M883	Chloride intracellular channel protein 2^b^
Q62770	Protein unc-13 homolog C^b^
Q63100	Cytoplasmic dynein 1 intermediate chain 1^b^
Q6IG00	Keratin, type II cytoskeletal 4^b^
Q8K3Y6	Zinc finger CCCH-type antiviral protein 1^b^
Q9R0L4	Cullin-associated NEDD8-dissociated protein 2^b^
O35816	Sodium myo-inositol transporter^b^
Q3B8P7	RCG58555, isoform CRA_a^b^
Q5I0E7	Transmembrane emp24 domain-containing protein 9^b^
Q5RK26	Polr3a protein^b^
Q7TPK7	Ac2-048^b^
Q80WM6	2',5'-oligoisoadenylate synthetase-dependent ribonuclease L^b^
**Q68FT5**^**a**^	**BHMT2 S-methylmethionine homocysteine S-methyltransferase** ^**b**^

^a^In bold, proteins selected for validation by immunoprecipitation.

^b^Proteins found in only one experiment, but that are related to BHMT protein function or subcellular localization.

In parallel, YTH screening of a rat liver cDNA library was performed for the identification of additional interaction targets using the full-length ORF of rat *Bhmt* as bait. This screening rendered 14 putative preys that grew in the–AHLW high stringency SC media (Table 5). Interestingly, seven of these putative preys were previously identified by AP-MS, namely S-methylmethionine homocysteine methyltransferase (BHMT2), transthyretin, 4-hydroxyphenylpyruvic acid dioxygenase (HPD), glutathione-S-transferase alpha type 2, alpha-2u globulin PGCL1/Major urinary protein, ornithine transcarbamylase and aldolase b protein (ALDOB). Among them, BHMT2 binds strongly to intein and intein-BHMT columns, but more peptides were recovered from peaks eluting from intein-BHMT (34 scans, 3 peptides) than from intein columns (18 scans, 2 peptides). Thus, the relevance of the BHMT-BHMT2 interaction was confirmed. Regarding the 6 remaining preys identified in the yeast two-hybrid screening and also found by AP-MS, all of them showed unspecific binding to chitin or intein columns and no peptide was found on intein-BHMT eluents, therefore their interaction with BHMT could not be validated.

**Table 5:** Hepatic BHMT interaction targets identified by yeast two-hybrid.

Protein code	Protein	gene	Clones found
P62982	Ubiquitin-40S ribosomal protein S27a	*Rps27a*	1
P02767	Transthyretin precursor (Prealbumin)	*Ttr*	1
P09367	serine dehydratase/Sds protein	*Sds*	2
P20673	argininosuccinate lyase	*Asl*	1
P05503	cytochrome c oxidase subunit I	*Mtco1*	2
P32755	4-hydroxyphenylpyruvic acid dioxygenase	*Hpd*	1
P04903	Glutathione-S-transferase, alpha type 2	*Gsta2*	1
P02761	alpha-2u globulin PGCL1/Major urinary protein	*-*	1
-	Similar to Hypothetical protein BC014729	*-*	1
Q6RJR6	reticulon 3 protein isoform b	*Rtn3*	1
P00481	ornithine transcarbamylase	*Otc*	2
P00884	Aldolase b protein	*Aldob*	1
Q68FT5	hypothetical protein LOC365972/S-methylmethionine homocysteine methyltransferase 2	*Bhmt2*	3
P02793	ferritin light chain subunit	*Ftl1*	1

**Fig 3:**
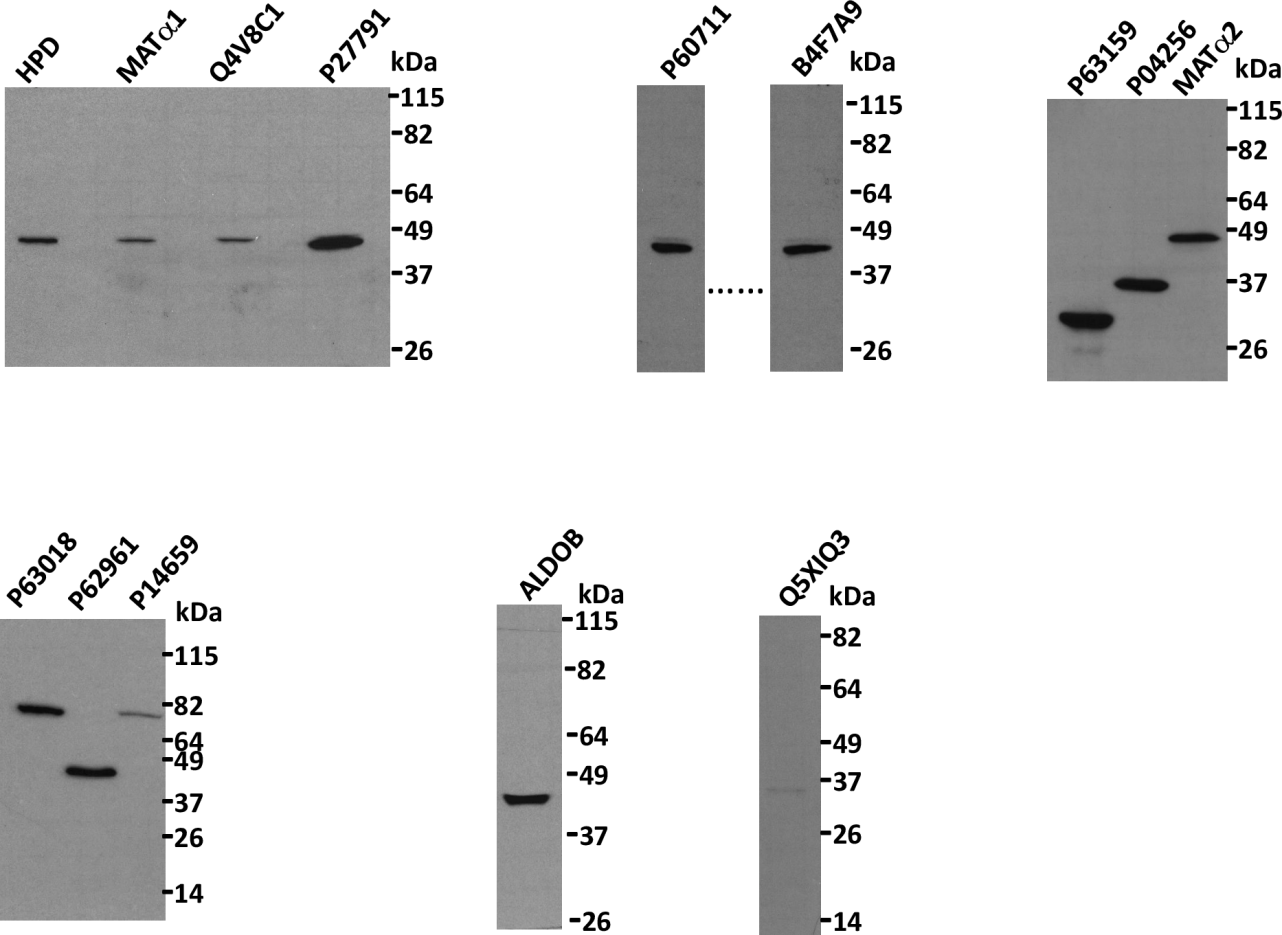
Expression of HA-tagged candidates for interaction with BHMT. Several proteins identified as potential targets for BHMT interaction during yeast two-hybrid and AP-MS screenings were cloned into pCMV-HA and expressed in several cell lines to verify their molecular size. The figure shows representative images of anti-HA immunoblots of those HA-tagged proteins exhibiting the correct size upon expression in Cos7 cells; the molecular weight of the standards is indicated on the side of each blot. HPD (P32755), 4-hydroxyphenylpyruvic acid dioxygenase; MATα1, methionine adenosyltransferase α1; Q4V8C1, RGD1306462 protein; P27791, cAMP-dependent protein kinase catalytic subunit alpha; P60711, actin cytoplasmic 1; B4F7A9, casein kinase 2 alpha2; P63159, high mobility group protein B1; P04256, heterogeneous nuclear ribonucleoprotein A1; MATα2, methionine adenosyltransferase α2; P63018, heat shock cognate 71 kDa protein; P62961, nuclease-sensitive element-binding protein 1; P14659, heat shock-related 70 kDa protein 2; ALDOB (P00884), aldolase B; Q5XIQ3, CXXC-type zinc finger protein 5.

Twenty additional proteins were selected from AP-MS data, together with MAT**α**1, MAT**α**2, HPD and ALDOB for further validation of their interaction with BHMT by coimmunoprecipitation. For this purpose, the ORFs of interest were cloned into pCMV4-HA for expression and the size of the tagged proteins verified by immunoblotting using anti-HA (Fig 3). Whenever possible, the ORFs corresponded to rat proteins, but there were cases in which only the human counterpart was available. This fact was not expected to exert a major drawback given the high identity level between rat and human BHMT sequences (~93%). Nevertheless, several proteins had to be excluded from further validation, as these HA-tagged preys did not achieve detectable expression levels in any of the cell lines used, or the size of the protein obtained differed notably from theoretical calculations. Therefore, only fourteen HA-tagged preys met the criteria for their use in coimmunoprecipitation experiments. Cotransfection with pFLAG-BHMT (47 kDa) and the pHA-prey plasmids of interest was then performed using different cell lines to ensure coexpression of both bait and prey. In several cases, expression and/or coexpression were not achieved, and hence validation of these interactions was precluded. Moreover, differences in the expression levels attained in lysates of cotransfectants and controls bearing a single plasmid were commonly observed independently of the pFLAG-BHMT/pHA-prey ratio used for transfection. These differences were taken into account for the analysis of immunoprecipitation results.

**Fig 4:**
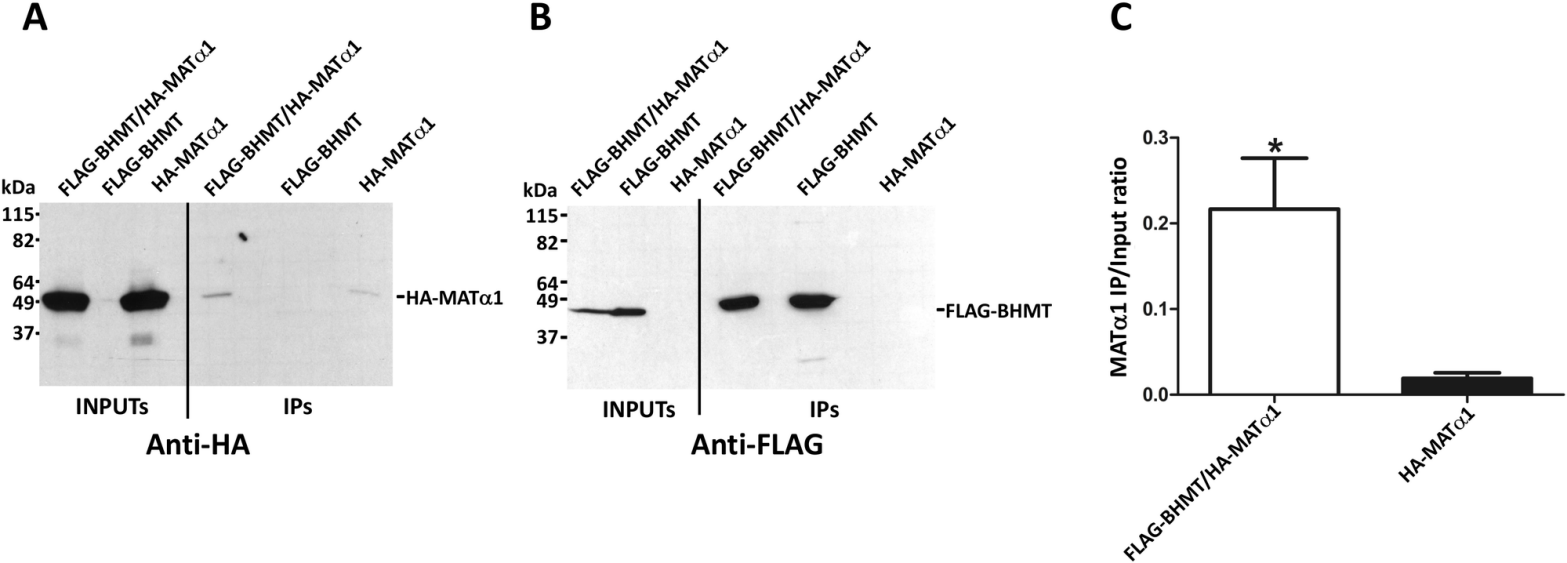
Validation of MATα1 as a BHMT interaction target. Cos7 cells were transfected with pFLAG-BHMT, pHA-MATα1 or cotransfected with both plasmids at a 1:1 (w/w) ratio. Lysates were obtained 48 hours posttransfection and immediately processed by anti-FLAG immunoprecipitation. Samples of the inputs (25 μl) and immunoprecipitates (40 μl) were analyzed by western blotting using specific antibodies and mouse TrueBlot. (A) Representative anti-HA immunoblots from a single immunoprecipitation experiment (N = 10). (B) Representative anti-FLAG results from a single immunoprecipitation experiment (N = 10). The size of the protein standards is indicated on the left side of each image. (C) Anti-HA signals were quantified using ImageJ and the immunoprecipitate/input ratio (mean ± SEM) for all the experiments calculated (N = 10) to correct for differences in expression between control bearing only pHA-MATα1 and cotransfectants. Statistical analysis of the data was performed by Student’s t-test using GraphPad Prism; *p≤0.05.

**Fig 5:**
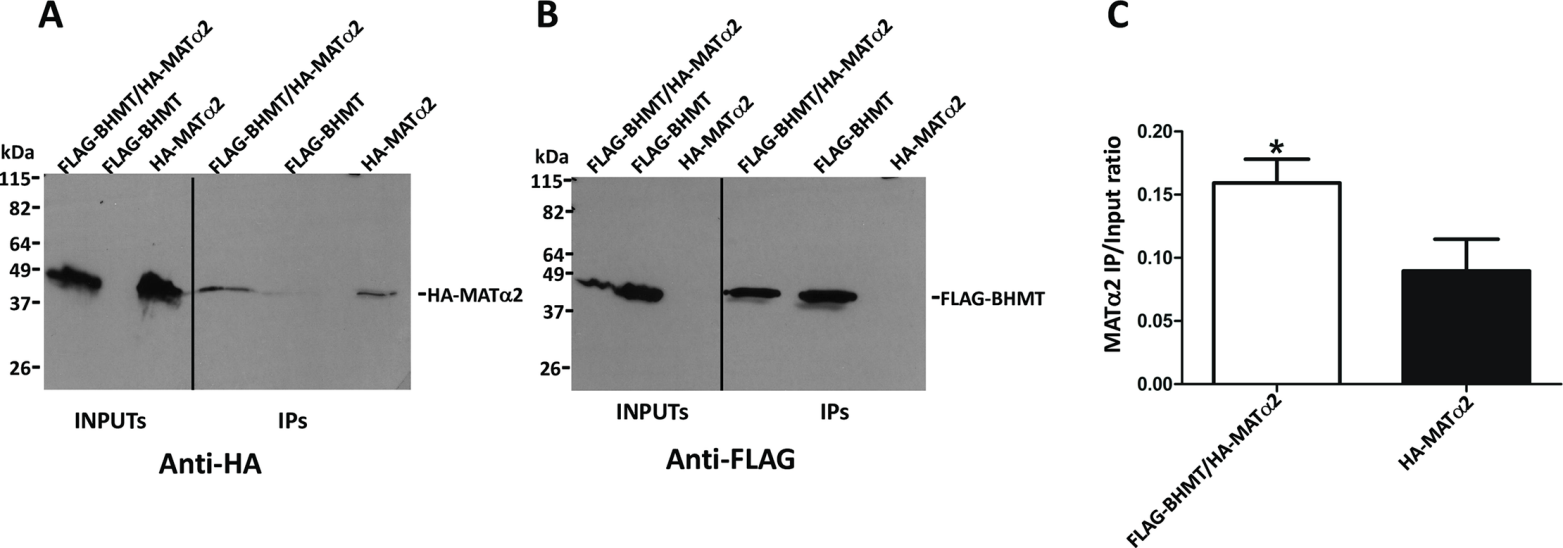
Confirmation of MATα2 and BHMT interaction. Cos7 cells were transfected with pFLAG-BHMT, pHA-MATα2 or cotransfected with both plasmids at a 1:1 (w/w) ratio. Lysates were obtained 48 hours posttransfection and immediately processed by anti-FLAG immunoprecipitation. Input (25 μl) and immunoprecipitate samples (40 μl) were analyzed by western blotting using specific antibodies and mouse TrueBlot. (A) Representative anti-HA results from a single immunoprecipitation experiment (N = 8). (B) Representative anti-FLAG immunoblot from a single immunoprecipitation experiment (N = 8). The size of the protein standards is indicated on the left side of each image. (C) Anti-HA signals were quantified using ImageJ and the immunoprecipitate/input ratio (mean ± SEM) for all the experiments calculated (N = 8) to correct for differences in expression between control bearing only pHA-MATα2 and cotransfectants. Statistical analysis of the data was performed by Student’s t-test using GraphPad Prism; *p≤0.05.

First, we concentrated on preys of the methionine cycle that were found by AP-MS, specifically the catalytic subunits of methionine adenosyltransferases (MATs), which are directly linked to BHMT function as consumers of methionine. Anti-FLAG immunoprecipitation from cotransfected cells allowed recovery of HA-MATα1, whereas a nonspecific background was found in immunoprecipitates of control cells overexpressing only HA-MATα1 (Fig 4). Changes in the composition of the buffers (e.g. salt concentration, detergents) did not reduce this background. Quantification of the HA-signals in the inputs and immunoprecipitates was carried out and their ratio (IP/input) calculated to correct for differences in expression between samples. This ratio was also significantly higher for coimmunoprecipitates, thus confirming the BHMT/MATα1 interaction. When the same procedure was used with HA-MATα2, coimmunoprecipitation with FLAG-BHMT was also detected, but a higher background was consistently observed, which is favored by the higher HA-MATα2 expression levels obtained in control cells (Fig 5). Nevertheless, the difference between the calculated IP/input ratios was significant, also validating the BHMT/MATα2 interaction.

**Fig 6:**
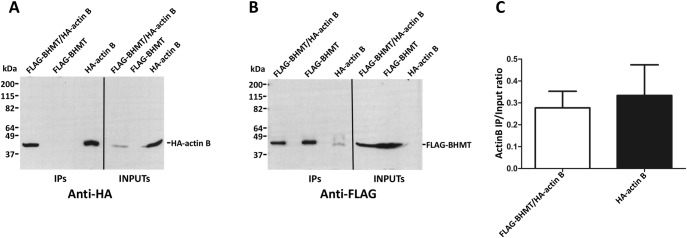
Corroboration of the BHMT/actin B interaction. Cos7 cells were transfected with pFLAG-BHMT, pHA-actin B or cotransfected with both plasmids at a 1:1 (w/w) ratio. Lysates were obtained 48 hours posttransfection and immediately processed by anti-FLAG immunoprecipitation. Input (25 μl) and immunoprecipitate samples (40 μl) were analyzed by western blotting using specific antibodies and mouse TrueBlot. (A) Representative anti-HA immunoblot from the immunoprecipitations experiments carried out (N = 7). (B) Representative anti-FLAG immunoprecipitations from the experiments performed (N = 7). The size of the protein standards is indicated on the left side of each image. (C) Anti-HA signals were quantified using ImageJ and the immunoprecipitate/input ratio (mean ± SEM) from all the experiments performed (N = 7) calculated to correct for differences in expression between control bearing only pHA-actin B and cotransfectants. Statistical analysis of the data was performed by Student’s t-test using GraphPad Prism; *p≤0.05.

**Fig 7:**
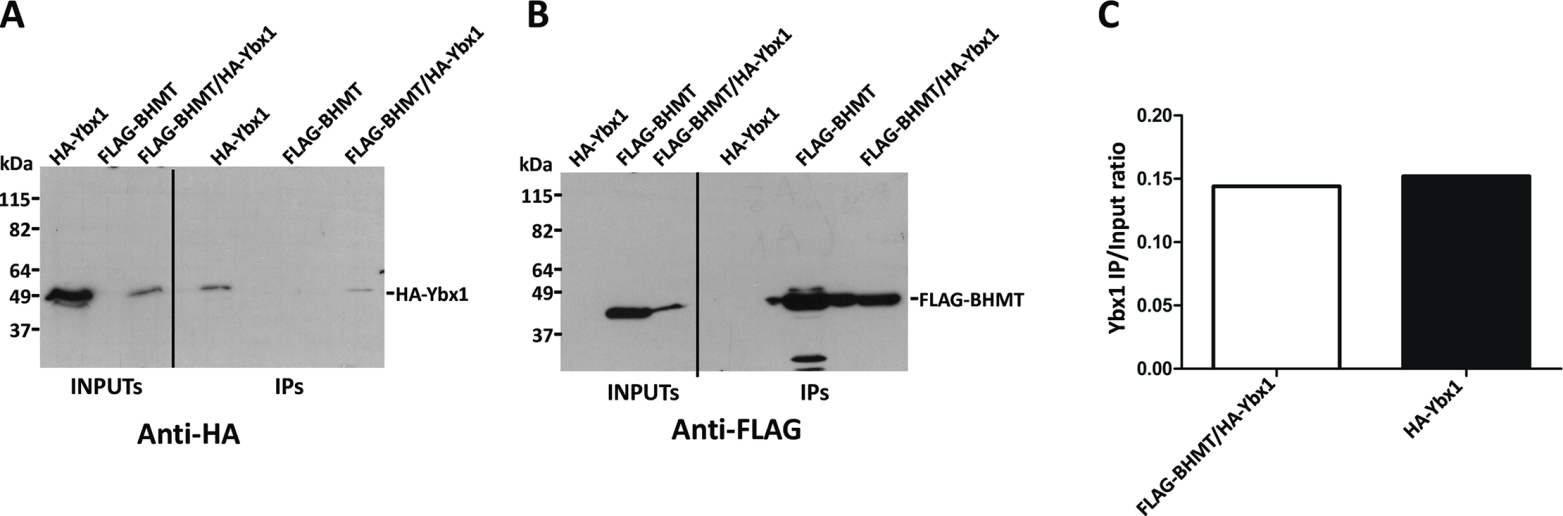
Testing Ybx1 as putative BHMT interaction target. Cos7 cells were transfected with pFLAG-BHMT, pHA-Ybx1 or cotransfected with both plasmids at a 1:1 (w/w) ratio. Lysates were obtained 48 hours posttransfection and immediately processed by anti-FLAG immunoprecipitation. Input (25 μl) and immunoprecipitate samples (40 μl) were analyzed by western blotting using specific antibodies and mouse TrueBlot. (A) Representative anti-HA images from the immunoprecipitation experiments carried out (N = 3). (B) Representative anti-FLAG immunoblot from the immunoprecipitation experiments performed (N = 3). The size of the protein standards is indicated on the left side of each image. (C) Anti-HA signals were quantified using ImageJ and the immunoprecipitate/input ratio (mean ± SEM) from all the experiments (N = 3) calculated to correct for differences in expression between control bearing only pHA-Ybx1 and cotransfectants. Statistical analysis of the data was performed by Student’s t-test using GraphPad Prism; *p≤0.05.

**Fig 8:**
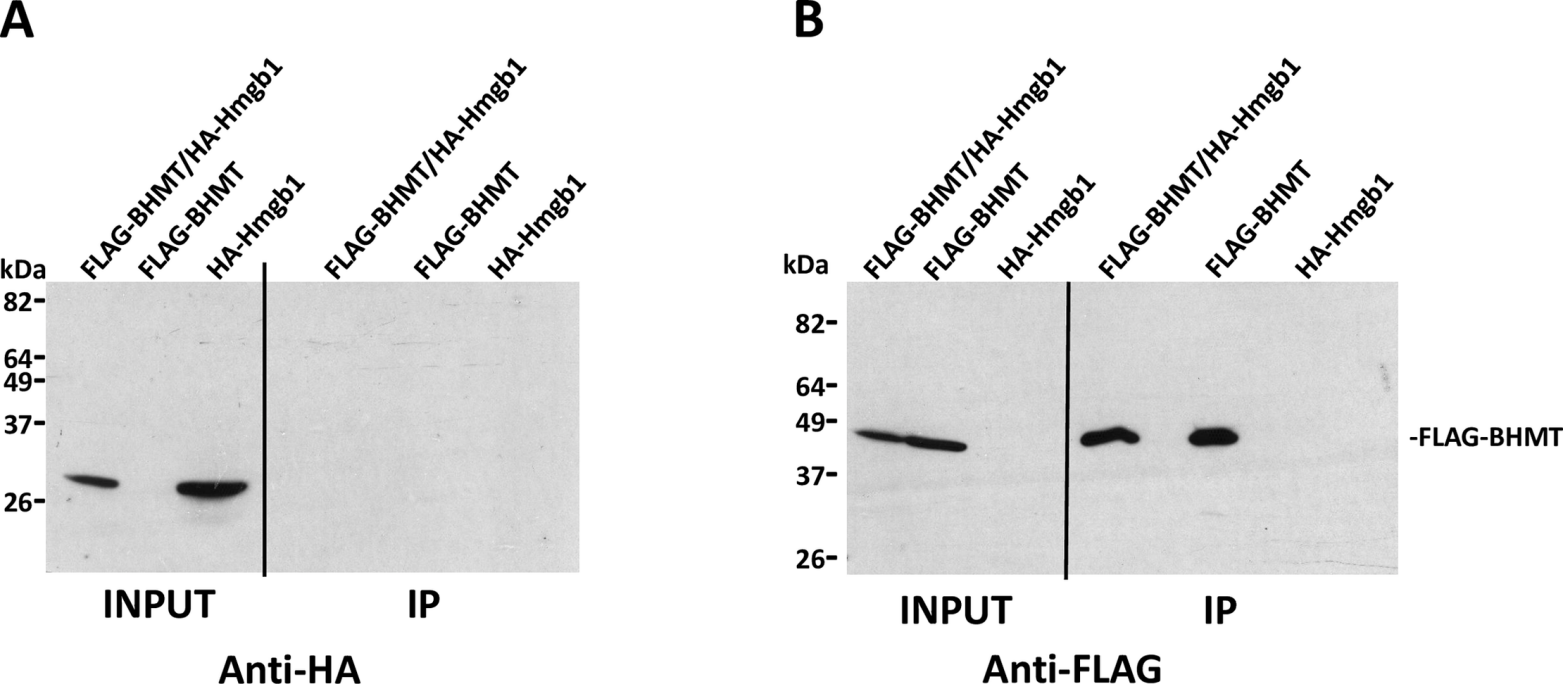
Validation of HMGB1 and BHMT interaction. Cos7 cells were transfected with pFLAG-BHMT, pHA-HMGB1 or cotransfected with both plasmids at a 1:1 (w/w) ratio. Lysates were obtained 48 hours posttransfection and immediately processed by anti-FLAG immunoprecipitation. Input (25 μl) and immunoprecipitate samples (40 μl) were analyzed by western blotting using specific antibodies and mouse TrueBlot. (A) Representative anti-HA immunoblots of the experiments performed (N = 3). (B) Representative anti-FLAG images of the experiments carried out (N = 3). The size of the protein standards is indicated on the left side of each image.

**Fig 9:**
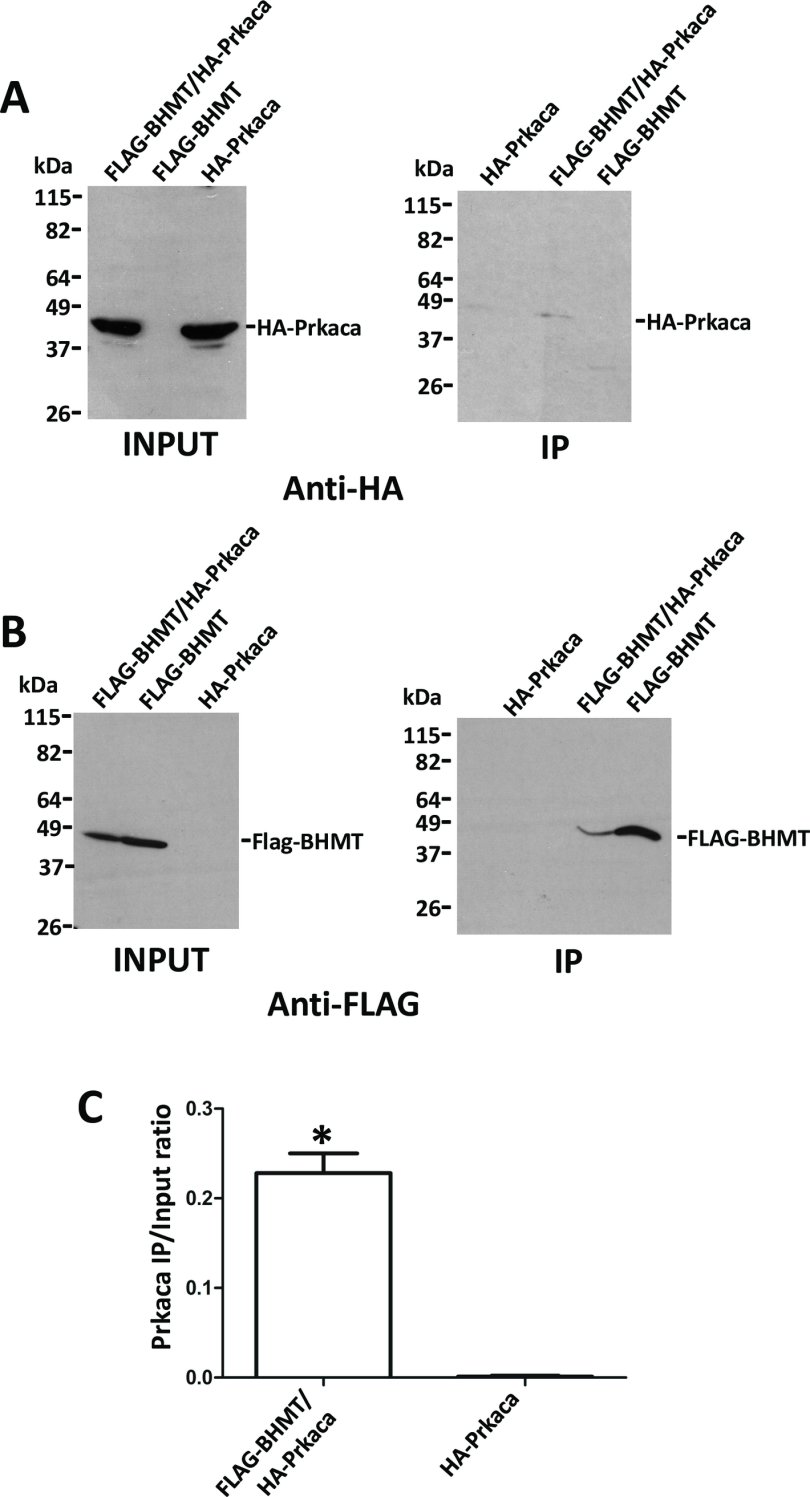
Coimmunoprecipitation of Prkaca and BHMT. Cos7 cells were transfected with pFLAG-BHMT, pHA-Prkaca or cotransfected with both plasmids at a 1:1 (w/w) ratio. Lysates were obtained 48 hours posttransfection and immediately processed by anti-FLAG immunoprecipitation. Input (25 μl) and immunoprecipitate samples (40 μl) were analyzed by western blotting mouse primary antibodies and mouse TrueBlot. (A) Representative anti-HA immunoblot of the immunoprecipitation experiments performed (N = 3). (B) Representative images of anti-FLAG immunoblots of the experiments carried out (N = 3). The size of the protein standards is indicated at the left side of each image. (C) Anti-HA signals were quantified from all experiments (N = 3) using ImageJ and the immunoprecipitate/input ratio (mean ± SEM) calculated to correct for differences in expression between control bearing only pHA-Prkaca and cotransfectants. Statistical analysis of the data was performed by Student’s t-test using GraphPad Prism; *p≤0.05.

Next, verification of the putative interaction between FLAG-BHMT and HA-tagged targets actin B (ACTB; P60711), nuclease-sensitive element-binding protein 1 (Ybx1; P62961), HMGB1 (P63159) and cAMP-dependent protein kinase catalytic subunit alpha (Prkaca; P27791) was carried out. Again, strong differences in expression levels were found between cotransfectants and controls carrying a single plasmid. Anti-FLAG and anti-HA immunoprecipitations were performed to analyze the putative interaction with HA-actin B due to the existence of unspecific signals from the anti-FLAG antibody. Nonspecific HA-actin B binding to FLAG-Sepharose reached similar levels than that detected in coimmunoprecipitates and no significant differences were found between the calculated IP/input ratios (Fig 6). Therefore, the BHMT/actin B interaction could not be confirmed. Similarly, coimmunoprecipitation of BHMT and Ybx1 was detected, but the high unspecific binding of Ybx1 precluded verification of this interaction (Fig 7). No HMGB1 signal was detected in control or coimmunoprecipitates, despite the existence of previous reports identifying this protein as a BHMT interaction partner (Fig 8). However, this result could be expected from the fact that the HMGB1-BHMT interaction requires translocation of HMGB1 from the nucleus to the cytoplasm, a process that was favored in previous reports. In contrast, HA-Prkaca coimunoprecipitated with FLAG-BHMT and no significant HA-signal was observed in the controls (Fig 9). Thus, the Prkaca was validated as a BHMT interactor.

**Fig 10:**
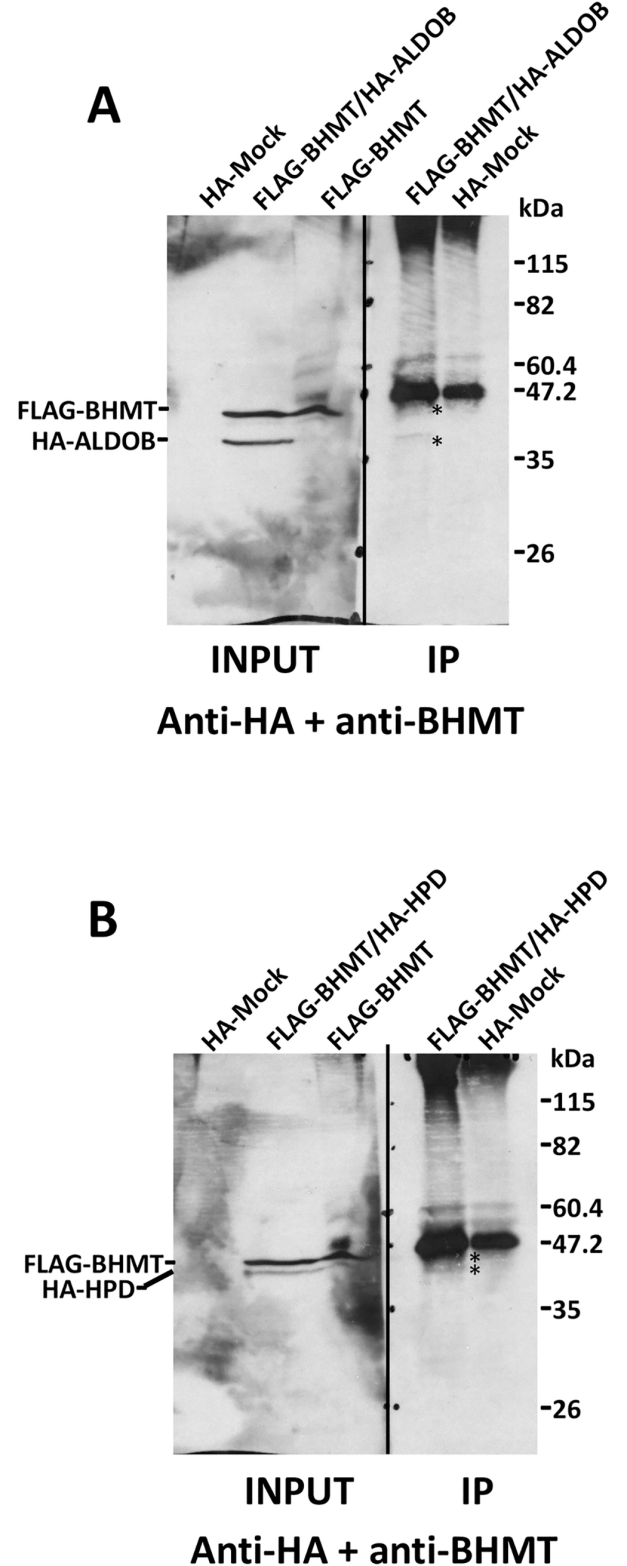
Confirmation of the BHMT/ALDOB and BHMT/HPD interaction. Cos7 cells were transfected with pFLAG-BHMT, pHA-ALDOB and pHA-HPD or cotransfected with pFLAG-BHMT and either of the pHA-preys at a 1:1 (w/w) ratio. Lysates were obtained 48 hours posttransfection and immediately processed by immunoprecipitation. Samples (30 μl) of the inputs and anti-HA immunoprecipitates were loaded on 14% SDS-PAGE gels and analyzed by western blotting. (A) Representative immunoblot of the immunoprecipitation experiments (N = 4) performed for ALDOB analyzed with a mixture of anti-HA and anti-BHMT. (B) Representative immunoblot of the immunoprecipitation experiments (N = 4) performed for HPD analyzed with a mixture of anti-HA and anti-BHMT. The size of the protein standards is indicated on the side of each image. (*) Specific BHMT, ALDOB and HPD bands are indicated in the immunoprecipitation lanes.

We also sought out for validation of putative preys found in the YTH screening that were detected in previous AP-MS experiments, but that showed unspecific binding to chitin columns. This is the case of ALDOB and HPD. For this purpose, cotransfection with pFLAG-BHMT and pHA-ALDOB or pHA-HPD was performed and anti-HA immunoprecipitation carried out (Fig 10). In both cases, anti-BHMT recognized a band immediately below the heavy chain of anti-HA in the immunoprecipitates of cotransfected cells. Thus, ALDOB and HPD were confirmed as BHMT interaction targets.

**Fig 11:**
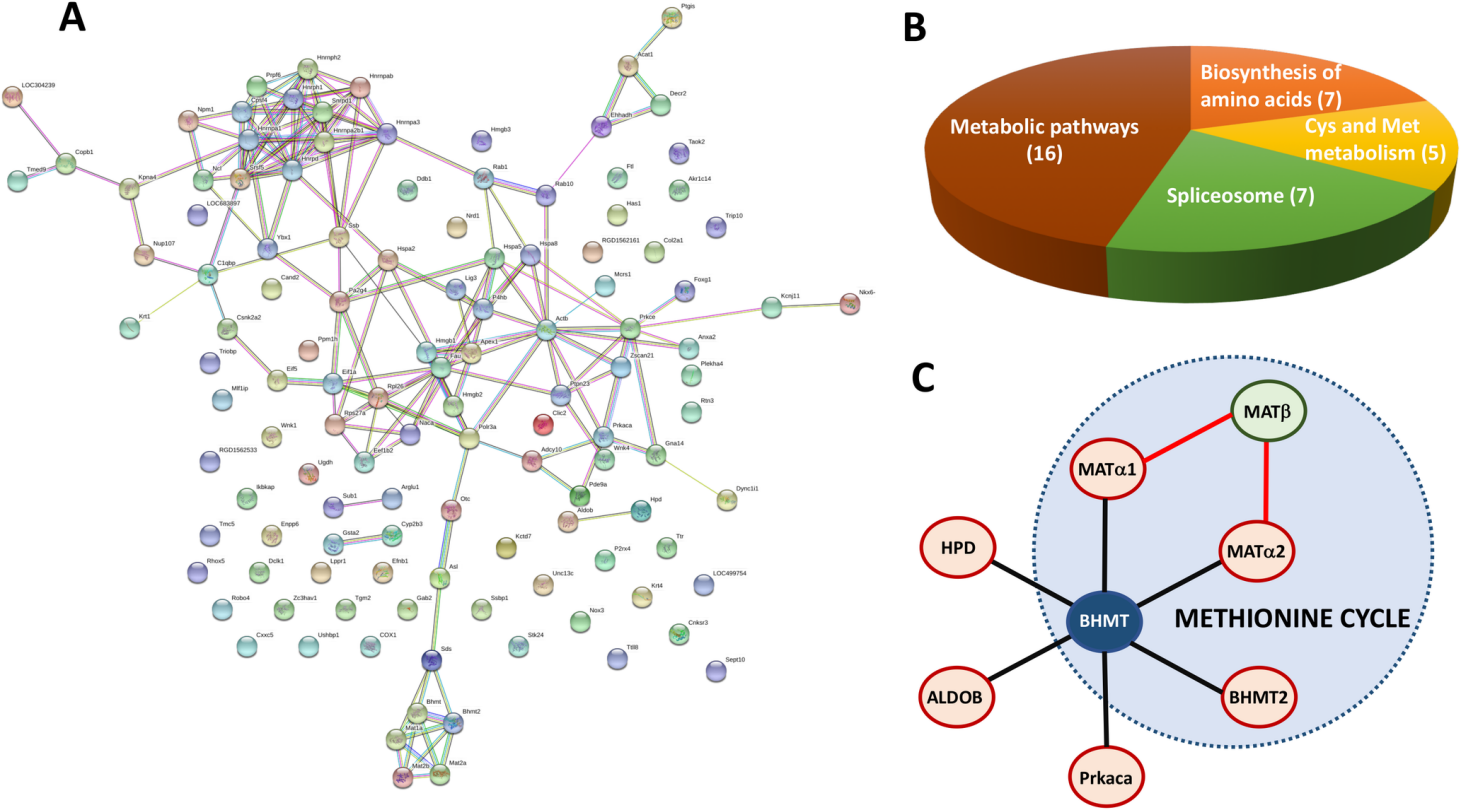
Network analysis using STRING and new validated interaction targets. The whole set of BHMT interaction targets identified by affinity purification/mass spectrometry and yeast two-hybrid was analyzed using STRING set at medium confidence 0.4. (A) The panel shows the network links. (B) Pie chart illustrating the distribution between KEGG pathways with false discovery rates <0.05; The number of proteins identified for each pathway is indicated in parenthesis. (C) Schematic representation of the validated BHMT interactions identified in the present study. Abbreviations: BHMT, betaine homocysteine S-methyltransferase; BHMT2, S-methylmethionine methyltransferase or betaine homocysteine methyltransferase 2; MATα1, methionine adenosyltransferase alpha1; MATα2, methionine adenosyltransferase alpha2; MATβ, methionine adenosyltransferase beta; ALDOB, aldolase b; Prkaca, cAMP-dependent protein kinase catalytic subunit alpha; HPD, 4-hydroxyphenylpyruvic acid dioxygenase.

Finally, protein-protein interaction network analysis was carried out for the whole set of BHMT interaction targets identified by AP-MS and YTH using STRING. The PPI network constructed contained 131 proteins, 122 nodes and 165 edges (Fig 11A). The average node degree was 2.7 and the PPI enrichment value 4.81e-05. This tool classified the targets in a large number of pathways with statistical significance (FDR <0.05). Several GO pathways were found and grouped according to the biological process (152; Table 6), the molecular function (48; Table 7) and the cellular component (58; Table 8). Moreover, a limited number of KEGG pathways (4) with FDR <0.05 were also identified, including the biosynthesis of amino acids (ID 01230; 4.63e-05), cysteine and methionine metabolism (ID 00270; 0.000212), the spliceosome (ID 03040; 0.000408) and metabolic pathways (ID 01100; 0.04) (Fig 11B). Some of these pathways were also recognized when analysis of the interaction targets was performed with Bioprofiling, although the set with statistical significance was more limited.

**Table 6:** STRING classification of BHMT interaction targets according to the biological process (GO).

pathway ID	pathway description	count in protein set	false discovery rate
GO:0008152	metabolic process	65	1.57e-18
GO:0044237	cellular metabolic process	60	1.57e-18
GO:0044238	primary metabolic process	59	1.13e-17
GO:0071704	organic substance metabolic process	60	1.13e-17
GO:0009987	cellular process	72	2.04e-17
GO:0006807	nitrogen compound metabolic process	45	1.32e-16
GO:0034641	cellular nitrogen compound metabolic process	42	1.86e-15
GO:0008150	biological_process	66	2.88e-12
GO:1901360	organic cyclic compound metabolic process	36	1.36e-11
GO:0044260	cellular macromolecule metabolic process	42	1.48e-11
GO:0006725	cellular aromatic compound metabolic process	34	6.32e-11
GO:0043170	macromolecule metabolic process	43	1.06e-10
GO:0046483	heterocycle metabolic process	33	1.94e-10
GO:0006139	nucleobase-containing compound metabolic process	32	2.02e-10
GO:0044699	single-organism process	54	3.76e-08
GO:0044271	cellular nitrogen compound biosynthetic process	26	5.02e-08
GO:0010467	gene expression	26	2.3e-07
GO:0009058	biosynthetic process	30	3.64e-07
GO:0044249	cellular biosynthetic process	29	4e-07
GO:0050896	response to stimulus	40	4e-07
GO:0044763	single-organism cellular process	49	6.11e-07
GO:1901576	organic substance biosynthetic process	29	8.02e-07
GO:0090304	nucleic acid metabolic process	24	1.39e-06
GO:0006575	cellular modified amino acid metabolic process	9	1.52e-06
GO:0006396	RNA processing	12	2.32e-06
GO:1901564	organonitrogen compound metabolic process	19	2.34e-06
GO:0006397	mRNA processing	10	2.89e-06
GO:0042221	response to chemical	29	3.4e-06
GO:0008380	RNA splicing	9	5.87e-06
GO:0051252	regulation of RNA metabolic process	21	8.69e-06
GO:0031323	regulation of cellular metabolic process	29	1.29e-05
GO:0044281	small molecule metabolic process	19	1.41e-05
GO:0060255	regulation of macromolecule metabolic process	28	1.51e-05
GO:0044767	single-organism developmental process	31	1.52e-05
GO:0019219	regulation of nucleobase-containing compound metabolic process	22	2.16e-05
GO:0080090	regulation of primary metabolic process	28	2.16e-05
GO:0051171	regulation of nitrogen compound metabolic process	23	2.51e-05
GO:0048856	anatomical structure development	28	3.69e-05
GO:0016070	RNA metabolic process	20	3.86e-05
GO:0044710	single-organism metabolic process	27	3.86e-05
GO:0006556	S-adenosylmethionine biosynthetic process	3	3.89e-05
GO:0019222	regulation of metabolic process	30	4.05e-05
GO:0065007	biological regulation	41	4.4e-05
GO:0048519	negative regulation of biological process	26	4.92e-05
GO:0050794	regulation of cellular process	38	7.24e-05
GO:0044267	cellular protein metabolic process	22	7.34e-05
GO:0051716	cellular response to stimulus	30	8.03e-05
GO:0048523	negative regulation of cellular process	24	0.000104
GO:0034654	nucleobase-containing compound biosynthetic process	18	0.000117
GO:0010468	regulation of gene expression	21	0.000126
GO:0010033	response to organic substance	23	0.000134
GO:2000112	regulation of cellular macromolecule biosynthetic process	20	0.000157
GO:0070887	cellular response to chemical stimulus	19	0.000164
GO:0050789	regulation of biological process	38	0.000191
GO:0007275	multicellular organismal development	26	0.00031
GO:0048731	system development	24	0.000343
GO:0019538	protein metabolic process	23	0.000393
GO:0044272	sulfur compound biosynthetic process	5	0.000456
GO:1901566	organonitrogen compound biosynthetic process	12	0.00052
GO:0034645	cellular macromolecule biosynthetic process	19	0.000618
GO:0048522	positive regulation of cellular process	24	0.000655
GO:0071310	cellular response to organic substance	16	0.000737
GO:1901699	cellular response to nitrogen compound	10	0.000862
GO:0006355	regulation of transcription, DNA-templated	17	0.00102
GO:0045934	negative regulation of nucleobase-containing compound metabolic process	11	0.0014
GO:1901698	response to nitrogen compound	14	0.00152
GO:0032501	multicellular organismal process	28	0.00209
GO:0001889	liver development	6	0.00211
GO:0030334	regulation of cell migration	9	0.00211
GO:0061008	hepaticobiliary system development	6	0.00211
GO:0051253	negative regulation of RNA metabolic process	10	0.00219
GO:0042398	cellular modified amino acid biosynthetic process	4	0.00224
GO:0006403	RNA localization	5	0.0023
GO:0010608	posttranscriptional regulation of gene expression	7	0.0025
GO:0042981	regulation of apoptotic process	13	0.0025
GO:0009719	response to endogenous stimulus	17	0.00251
GO:0009056	catabolic process	14	0.00253
GO:0071417	cellular response to organonitrogen compound	9	0.00259
GO:0048513	organ development	18	0.0026
GO:0010629	negative regulation of gene expression	11	0.00291
GO:0033528	S-methylmethionine cycle	2	0.00326
GO:0042450	arginine biosynthetic process via ornithine	2	0.00326
GO:0042493	response to drug	11	0.00326
GO:0019752	carboxylic acid metabolic process	11	0.00329
GO:0010243	response to organonitrogen compound	13	0.00338
GO:1901701	cellular response to oxygen-containing compound	11	0.00364
GO:2000113	negative regulation of cellular macromolecule biosynthetic process	10	0.00367
GO:0072521	purine-containing compound metabolic process	7	0.00393
GO:0043412	macromolecule modification	16	0.00406
GO:0048518	positive regulation of biological process	24	0.00406
GO:0010605	negative regulation of macromolecule metabolic process	14	0.00408
GO:0080134	regulation of response to stress	10	0.00442
GO:0014070	response to organic cyclic compound	14	0.0045
GO:0055086	nucleobase-containing small molecule metabolic process	8	0.005
GO:0009605	response to external stimulus	15	0.0051
GO:0046683	response to organophosphorus	6	0.00524
GO:0044707	single-multicellular organism process	26	0.00548
GO:0044248	cellular catabolic process	12	0.00562
GO:1901575	organic substance catabolic process	12	0.00567
GO:0006950	response to stress	19	0.00599
GO:0055114	oxidation-reduction process	11	0.00649
GO:0035556	intracellular signal transduction	12	0.00651
GO:1903311	regulation of mRNA metabolic process	4	0.00665
GO:0006464	cellular protein modification process	15	0.00674
GO:0032879	regulation of localization	17	0.00674
GO:1903312	negative regulation of mRNA metabolic process	3	0.0069
GO:1990267	response to transition metal nanoparticle	6	0.00723
GO:0006796	phosphate-containing compound metabolic process	14	0.0073
GO:0006351	transcription, DNA-templated	13	0.0085
GO:0006810	transport	21	0.00895
GO:0007399	nervous system development	15	0.00983
GO:1901700	response to oxygen-containing compound	15	0.00988
GO:0014074	response to purine-containing compound	6	0.0103
GO:0071345	cellular response to cytokine stimulus	7	0.0105
GO:0006730	one-carbon metabolic process	3	0.0106
GO:0043488	regulation of mRNA stability	3	0.0106
GO:0009725	response to hormone	13	0.011
GO:0010038	response to metal ion	8	0.0115
GO:0071495	cellular response to endogenous stimulus	11	0.0115
GO:0009066	aspartate family amino acid metabolic process	3	0.012
GO:0043604	amide biosynthetic process	7	0.0121
GO:0045787	positive regulation of cell cycle	5	0.0123
GO:0071786	endoplasmic reticulum tubular network organization	2	0.0125
GO:0031324	negative regulation of cellular metabolic process	13	0.0134
GO:0034097	response to cytokine	8	0.0135
GO:1903034	regulation of response to wounding	6	0.0135
GO:0048583	regulation of response to stimulus	16	0.0151
GO:0051260	protein homooligomerization	6	0.0167
GO:0071375	cellular response to peptide hormone stimulus	6	0.0177
GO:0043434	response to peptide hormone	8	0.0183
GO:0010035	response to inorganic substance	9	0.0191
GO:0032069	regulation of nuclease activity	2	0.0191
GO:0050658	RNA transport	4	0.0191
GO:0023051	regulation of signaling	15	0.0206
GO:0045892	negative regulation of transcription, DNA-templated	8	0.0211
GO:0051726	regulation of cell cycle	8	0.0211
GO:0010646	regulation of cell communication	15	0.0227
GO:0046128	purine ribonucleoside metabolic process	5	0.0236
GO:1901605	alpha-amino acid metabolic process	5	0.0244
GO:0051179	localization	22	0.0272
GO:0044711	single-organism biosynthetic process	10	0.0286
GO:0065003	macromolecular complex assembly	10	0.0314
GO:0071840	cellular component organization or biogenesis	20	0.0319
GO:0051259	protein oligomerization	7	0.032
GO:0000050	urea cycle	2	0.0351
GO:0071316	cellular response to nicotine	2	0.0351
GO:1901135	carbohydrate derivative metabolic process	8	0.0368
GO:0006417	regulation of translation	5	0.0369
GO:0032414	positive regulation of ion transmembrane transporter activity	3	0.0386
GO:0006461	protein complex assembly	9	0.0403
GO:0070271	protein complex biogenesis	9	0.0403
GO:0071407	cellular response to organic cyclic compound	7	0.0426

**Table 7:** STRING classification of BHMT interaction targets according to molecular function (GO).

pathway ID	pathway description	count in protein set	false discovery rate
GO:1901363	heterocyclic compound binding	47	1.69e-15
GO:0097159	organic cyclic compound binding	47	1.71e-15
GO:0003674	molecular_function	69	4.1e-14
GO:0005488	binding	59	8.46e-12
GO:0003723	RNA binding	22	9.75e-12
GO:0043167	ion binding	44	1.15e-10
GO:0003676	nucleic acid binding	28	2.69e-10
GO:0000166	nucleotide binding	29	3.54e-10
GO:0036094	small molecule binding	31	3.54e-10
GO:0003697	single-stranded DNA binding	7	3.31e-08
GO:0043168	anion binding	28	7.59e-08
GO:0008134	transcription factor binding	10	5.78e-07
GO:0005515	protein binding	32	1.27e-06
GO:0032550	purine ribonucleoside binding	20	8.44e-06
GO:0035639	purine ribonucleoside triphosphate binding	20	8.44e-06
GO:0097367	carbohydrate derivative binding	22	8.44e-06
GO:0003824	catalytic activity	34	1.04e-05
GO:0032555	purine ribonucleotide binding	20	1.04e-05
GO:0043566	structure-specific DNA binding	8	1.64e-05
GO:0005524	ATP binding	17	2.62e-05
GO:0044822	poly(A) RNA binding	11	4.59e-05
GO:0003677	DNA binding	15	5.7e-05
GO:0019899	enzyme binding	14	7.11e-05
GO:0043169	cation binding	25	0.000118
GO:0046872	metal ion binding	24	0.000199
GO:0008289	lipid binding	10	0.00102
GO:0016491	oxidoreductase activity	11	0.00126
GO:0016829	lyase activity	6	0.00147
GO:0005198	structural molecule activity	9	0.00152
GO:0043021	ribonucleoprotein complex binding	4	0.00188
GO:0004478	methionine adenosyltransferase activity	2	0.00229
GO:0044877	macromolecular complex binding	12	0.00229
GO:0047150	betaine-homocysteine S-methyltransferase activity	2	0.00229
GO:0008898	S-adenosylmethionine-homocysteine S-methyltransferase activity	2	0.00667
GO:0051082	unfolded protein binding	3	0.00877
GO:0097100	supercoiled DNA binding	2	0.0124
GO:0003729	mRNA binding	4	0.0132
GO:0016740	transferase activity	14	0.0174
GO:0042162	telomeric DNA binding	2	0.0193
GO:0016860	intramolecular oxidoreductase activity	3	0.0266
GO:0008301	DNA binding, bending	2	0.0379
GO:0016840	carbon-nitrogen lyase activity	2	0.0379
GO:0043565	sequence-specific DNA binding	7	0.0385
GO:0019901	protein kinase binding	6	0.0415
GO:0051059	NF-kappaB binding	2	0.0476
GO:0004672	protein kinase activity	7	0.0491
GO:0004674	protein serine/threonine kinase activity	6	0.0495
GO:0019843	rRNA binding	3	0.0497

**Table 8:** STRING classification of BHMT interaction targets according to the cellular component (GO).

Cellular component (GO)			
pathway ID	pathway description	count in protein set	false discovery rate
GO:0044424	intracellular part	76	2.91e-20
GO:0005622	intracellular	76	5.88e-20
GO:0005737	cytoplasm	69	1.93e-17
GO:0044464	cell part	76	1.93e-17
GO:0005623	cell	76	1.94e-17
GO:0005634	nucleus	46	5.75e-15
GO:0005575	cellular_component	74	2.38e-14
GO:0043226	organelle	63	2.38e-14
GO:0044446	intracellular organelle part	50	3.06e-14
GO:0032991	macromolecular complex	41	2.36e-13
GO:0043229	intracellular organelle	60	4.26e-13
GO:0043227	membrane-bounded organelle	58	1.01e-12
GO:0043231	intracellular membrane-bounded organelle	55	1.42e-11
GO:0044428	nuclear part	26	1.4e-09
GO:0070013	intracellular organelle lumen	26	2.16e-09
GO:0044444	cytoplasmic part	46	2.18e-09
GO:0030529	ribonucleoprotein complex	15	4.57e-09
GO:0043232	intracellular non-membrane-bounded organelle	29	4.57e-09
GO:0031981	nuclear lumen	22	3.42e-08
GO:0048471	perinuclear region of cytoplasm	13	4.19e-06
GO:0070062	extracellular exosome	18	7.02e-06
GO:0005829	cytosol	20	9.86e-06
GO:0031988	membrane-bounded vesicle	22	1.03e-05
GO:0043234	protein complex	26	1.51e-05
GO:0044421	extracellular region part	22	1.51e-05
GO:0031982	vesicle	22	2.21e-05
GO:0016020	membrane	41	2.31e-05
GO:0005681	spliceosomal complex	6	3.33e-05
GO:0005654	nucleoplasm	15	3.77e-05
GO:0005576	extracellular region	23	0.00014
GO:0005739	mitochondrion	15	0.000765
GO:0000793	condensed chromosome	5	0.000945
GO:0048269	methionine adenosyltransferase complex	2	0.000945
GO:0098588	bounding membrane of organelle	17	0.000945
GO:0031090	organelle membrane	20	0.000973
GO:0005793	endoplasmic reticulum-Golgi intermediate compartment	4	0.00136
GO:0012505	endomembrane system	21	0.00281
GO:0005730	nucleolus	8	0.00313
GO:0005783	endoplasmic reticulum	13	0.00434
GO:1990124	messenger ribonucleoprotein complex	2	0.00474
GO:0005694	chromosome	7	0.00912
GO:0043209	myelin sheath	5	0.00912
GO:0005856	cytoskeleton	12	0.0138
GO:0042470	melanosome	4	0.0138
GO:0016607	nuclear speck	4	0.0146
GO:0034663	endoplasmic reticulum chaperone complex	2	0.0146
GO:0042175	nuclear outer membrane-endoplasmic reticulum membrane network	9	0.0183
GO:0005886	plasma membrane	22	0.0188
GO:0044451	nucleoplasm part	6	0.0225
GO:0000777	condensed chromosome kinetochore	3	0.0226
GO:0070852	cell body fiber	2	0.0226
GO:0071944	cell periphery	22	0.0237
GO:0044427	chromosomal part	6	0.0261
GO:0044432	endoplasmic reticulum part	9	0.0272
GO:0000779	condensed chromosome, centromeric region	3	0.0328
GO:0042995	cell projection	14	0.0332
GO:0044430	cytoskeletal part	9	0.0447
GO:0044431	Golgi apparatus part	7	0.0499

## Discussion

The association of hyperhomocysteinemia (HHcy) with a large variety of diseases has increased the interest in the regulation of enzymes involved in Hcy metabolism and, among them BHMT. Knowledge about this protein and its regulation was hampered by a number difficulties since its discovery approximately 70 years ago (reviewed in [6]). Nevertheless, the last decades have seen a notable increase in data regarding the role of BHMT in disease. Several high throughput studies have provided information concerning PTMs occurring on BHMT or its putative interaction partners in human, rat and mouse [26–36, 49]. These targets are expected to be conserved among species given the high level of sequence identity (>90%) shown by BHMTs. However, the major drawback of these studies relies on the fact that only a few of them have gone further to confirm the interactions detected using additional techniques.

BHMT interaction targets listed in the IntAct and BioGRID databases, as well as in the literature include approximately 22 interaction partners (Table 9) [12, 33–36, 50–57]. However, only seven of these targets have been also identified in our study. Moreover, our results rise doubts about the validity of several BHMT interaction partners included in this list, as tubulin, 10-formyltetrahydrofolate dehydrogenase (Aldh1l1) and carbamoyl phosphate synthetase 1 show unspecific binding to chitin and chitin-intein columns, and no interaction with chitin-intein-BHMT beads. Therefore, among the previously reported BHMT interaction targets only 14-3-3 protein epsilon (Ywhae), tissue-type transglutaminase [33, 51], HMGB1 [36], and BHMT2 [34, 35] bind to BHMT columns in our study. These interactions exhibit different characteristics. For example, the Ywhae-BHMT interaction is lost in the presence of low salt concentrations, whereas the BHMT2-BHMT interaction remains stable. Furthermore, the Ywhae-BHMT interaction is detected only in one of our AP-MS experiments, suggesting that the interaction might be very weak or take place only under specific conditions.

**Table 9:** Interactions reported for BHMT in human, rat or mouse.

target	Uniprot code	method	organism	reference
ApoB mRNA editing		Screening of liver cDNA expression library	rat	[12]
Myotubularin-related protein 6 (MTMR6)	Q9Y217	Affinity purification and MS^a^	human	[35]
High mobility group protein B1 (HMGB1)	P63159	Ischemia/reperfusion, liver IP^b^, 2D^c^, MS^a^	rat	[36]
Tubulin	P68370	Liver purification, SDS-PAGE and MS^a^	rat	[50]
Tissue-type transglutaminase	Q9WVJ6	*In vitro* modification and MS^a^	mouse, guinea pig, rat	[33, 51]
S-protein of HBV		YTH^d^ (liver library)	human	[52]
Glucocortocoid receptor (Nr3c1)	P06536	Liver immunoaffinity chromatography, 2D^c^, MS^a^	rat	[53]
10-formyltetrahydrofolate dehydrogenase + carbamoyl phosphate synthetase 1	P28037 P07756	Liver affinity purification and N-terminal sequencing	rat	[54]
Bardet-Biedl syndrome 1 protein (BBS1)	Q8NFJ9	YTH^d^ (fetal kidney library)	human	[55]
Bardet-Biedl syndrome 2 protein (BBS2)	Q9BXC9	YTH^d^ (fetal kidney library)	human	[55]
Bardet-Biedl syndrome 4 protein (BBS4)	Q96RK4	YTH^d^ (fetal kidney library)	human	[55]
Regulator complex protein LAMTOR3/ MAPKSP1	Q9UHA4	YTH^d^ (adult liver library)	human	[56]
Alpha-1,2-mannosyltransferase ALG9	Q9H6U8	YTH^d^ (adult liver library)	human	[56]
X antigen binding protein 1 of HBV/LAMTOR5	O43504	YTH^d^ (cDNA liver library)	human	[57]
S-methylmethionine homocysteine S-methyltransferase BHMT2	Q9H2M3	Affinity purification and MS^a^	human	[34, 35]
E3 ubiquitin-protein ligase ZNRF1	Q8ND25	Affinity purification and MS^a^	human	[34]
Melanoregulain (MREG)	Q8N565	Affinity purification and MS^a^	human	[34]
Aldehyde dehydrogenase family 16 member A1 (Aldh16a1)	Q3T1L0	Blue native gels	rat	IntAct
14-3-3 protein epsilon (Ywhae)	P62259	cosedimentation	mouse	IntAct
6-phosphoglucono lactone (Pgls)	Q9CQ60	cosedimentation	mouse	IntAct
3 beta-hydroxysteroid dehydrogenase type 7 (Hsd3b7)	Q9EQC1	cosedimentation	mouse	IntAct

^a^MS, mass spectrometry

^b^IP, immunoprecipitation

^c^2D, two-dimensional electrophoresis

^d^YTH, yeast two-hybrid

Regarding tissue-type transglutaminase, its interaction with BHMT was expected, since previous studies showed the ability of this enzyme to modify BHMT on a single peptide [33, 51]. This peptide contains four glutamine residues that can be transglutaminated by their crosslinking with lysine residues of other proteins or with free amines. Different degrees of modification can be detected in this peptide, but in all the cases transglutamination reduces BHMT activity [51].

The BHMT interaction with HMGB1 requires translocation of the latter from the nucleus to the cytoplasm, as demonstrated by coimmunoprecipitation from selective hepatic ischemia/reperfusion samples [36]. However, a small amount of HMGB1 seems to exist in the cytoplasm of control liver samples, as shown in the corresponding coimmunoprecipitates. Our AP-MS study confirms the existence of the HMGB1-BHMT interaction in normal liver, but no coimmunoprecipitation is detected in validation experiments. This opposite behavior could be explained by differences in the cytosolic samples used for coimmunoprecipitation experiments by both groups and may regard on disparities in the content or the presence/loss of specific PTMs in HMGB1. Precisely, Zhang et al. used control liver lobes obtained during a selective ischemia/reperfusion procedure to prepare their cytosolic samples [36], whereas our validation experiments were carried out with cytosol from cotransfected cell lines.

The BHMT-BHMT2 interaction was previously detected in two AP-MS high-throughput studies carried out with human samples [34, 35], and we found this interaction both in our AP-MS and YTH experiments. Thus, the validity of this interaction was confirmed. Moreover, our data indicate that this interaction is strong and/or favored in the presence of salt, since excision of the intein tag is required for BHMT2 elution from the chitin-intein-BHMT column. This fact could be expected from the high percentage of identity between both proteins (73%) and their similar structural features, with the exception of the C-terminal α-helix [58, 59]. Lack of this α-helix results in a reduced stability of the BHMT2 oligomer [24, 59], and hence under certain circumstances a heterotetrameric BHMT-BHMT2 association could be an advantage. Both proteins, BHMT and BHMT2 belong to the methionine cycle and, in their tetrameric form, catalyze the methylation of Hcy for the synthesis of methionine using different methyl donors [60]. While BHMT utilizes betaine for the synthesis of one molecule of methionine, BHMT2 uses S-methylmethionine to produce two molecules of this amino acid [6]. Therefore, a BHMT-BHMT2 heterotetramer may provide additional stability to BHMT2 that putatively results in an increased production of methionine.

Data of our AP-MS study further expand this BHMT interaction network including 128 new putative interaction partners, some of which have been validated by coimmunoprecipitation. Interestingly, two of the validated interaction targets are the catalytic subunits of methionine adenosyltransferases MATα1 and MATα2, which also belong to the methionine cycle. The function of these proteins also depends on their oligomerization that occurs into homodimers and homotetramers for MATα1 or homotetramers and heterotrimers for MATα2 [38, 61–63]. The resulting isoenzymes require a constant methionine supply for the synthesis of the main cellular methyl donor, S-adenosylmethionine [64]. If methionine levels are reduced, the need of this essential amino acid could be supported by Hcy recycling through methylation [6, 64]. It is under these circumstances when the interaction between BHMT and MATs could improve S-adenosylmethionine synthesis to sustain the large variety of reactions that depend on this key metabolite.

Another validated BHMT interaction target is Prkaca, the cAMP-dependent protein kinase catalytic subunit alpha, which phosphorylates a large number of substrates and is involved in key regulatory mechanisms [65]. Several high-throughput studies have analyzed the human, mouse and rat phosphoproteome and the results obtained indicate that BHMTs of these organisms can be phosphorylated on several residues [26, 49, 66–68]. Among them, some serine residues are predicted as putative targets for PKA phosphorylation by KinasePhos (S^330^ and S^368^) and NetPhos 3.1 (S^68^, S^222^, S^245^ and S^405^). Nevertheless, effects derived from BHMT phosphorylation have not been further explored, despite their putative involvement in the control of enzyme activity and/or association events. Additionally, phosphorylation could be implicated in the nucleocytoplasmic shuttling of BHMT, an event that to date has been only linked to changes in the GSH/GSSG ratio that occur in acute liver injury [11]. Combination of different PTMs, their sequential or independent incorporation may regulate binding to importin or exportin carriers and, in turn, the changes in subcellular distribution.

Additional BHMT interaction targets identified and validated in our study are ALDOB and HPD. The former is a glycolytic enzyme that synthesizes glyceraldehyde 3-phosphate from fructose 1,6-bisphosphate [69], whereas the latter is a key enzyme in phenylalanine and tyrosine degradation that converts 4-hydroxyphenylpyruvate in homogentisate [70]. Although no obvious link between these enzymes and BHMT is known, it is interesting to recall that fumaryl acetoacetate, a downstream metabolite of homogentisate, is an inhibitor of MAT [71]. Hence, interaction of HPD and BHMT may result in a crosstalk between both pathways in order to control methionine levels, which may otherwise increase in parallel with fumaryl acetoacetate concentrations as observed in tyrosinemia type I [72]. Indirect links between ALDOB and BHMT could derive from the following facts: i) NADP^+^ production in the polyol pathway controls S-adenosylmethionine synthesis through MAT II hetero-oligomerization, in turn, reducing methionine pools; ii) elimination of fructose, the final product of the polyol pathway, involves ALDOB; iii) among the downstream metabolites generated during fructose metabolism is acyl-glycerol, which joins apolipoproteins to form lipoproteins; and iv) apoB mRNA levels correlate with BHMT protein levels and increase when the latter is induced [12, 73]. Nevertheless, this putative crossregulation needs to be further explored.

A recent integration of over 9000 MS experiments in order to build a global map of human protein complexes, the hu.MAP [74], has shown BHMT only in two complexes. Complex number 25 comprises BHMT and BHMT2, whereas complex number 2283 contains BHMT, BHMT2 and tubulin-specific chaperone E (TBCE). The scores calculated for the complexes TBCE-BHMT2 (0.9388) and TBCE-BHMT (0.0953) yet suggest that binding to the chaperone occurs indirectly through BHMT2. These results are in accordance with our present study, where the BHMT-BHMT2 interaction is detected by AP-MS and YTH. Moreover, no BHMT-tubulin interaction is identified, further confirming the lack of specific binding observed in our AP-MS experiments, despite the previous proposal of BHMT coassembly with tubulin dimers into microtubules [50]. These differences may rely on the use of normal or taxol-treated rat liver samples and from the different techniques utilized in both studies, AP-MS or cosedimentation.

Network analysis shows three highly connected subnetworks within the whole dataset identified by AP-MS and YTH (Fig 11A). One of these groups comprises targets of the methionine cycle, a second is established around ACTB on one side and the ribosomal Fau protein on the other, and a third is centered around heterogeneous nuclear ribonucleoproteins (Hnrnp). Direct interactions of BHMT are only shown for partners within the methionine cycle and L-serine dehydratase/L-threonine deaminase (Sds; P09367), the latter starting the array of connections linking this pathway and other putative BHMT targets. While interactions within the methionine cycle may facilitate metabolite channeling through enzymes of this route, the BHMT-Sds interaction may result in a crosstalk between methionine synthesis and gluconeogenesis, putatively controlling the use of the amino acid for the latter when its levels are low. Only four KEGG pathways were enriched in this network analysis and all are related to metabolic processes, mainly amino acid metabolic routes (Fig 11B). Results of the cellular component show distribution of the interaction targets among the cytoplasm and other subcellular locations, where the presence of BHMT has not been verified or just recently detected, i.e. the nucleus [11]. Therefore, the classical view of BHMT as a cytoplasmic protein should no longer impede the analysis of its putative interactions out of this compartment that, in fact, should be pursued.

The interactions described in the present work may be involved directly or indirectly in HHcy, a condition related to a large variety of diseases that affect tissues with high (e.g. the liver) to undetectable (e.g. brain) BHMT levels [11], and for which impaired hepatic Hcy metabolism is the main contributor. Therefore, increases in total plasma Hcy levels may not always correlate with changes in expression, protein or activity levels of BHMT in the affected tissue, but rather reflect hepatic alterations due to different types of injury (i.e. alcohol-induced cirrhosis and drug ingestion), stress (i.e. oxidative stress) or deficiencies in essential nutrients (i.e. B-vitamins). Changes in Hcy metabolism of non-genetic origin commonly result from decreased expression of key enzymes of this pathway, including BHMT as observed in hepatoma, cirrhosis or acute liver injury [6, 37, 75]. Moreover, this reduced expression correlates with a decrease in cytoplasmic protein levels, putatively changing the hepatic BHMT interaction network. In this line, interactions with MATs or BHMT2 may be favored in the context of HHcy in an attempt to decrease cellular Hcy levels through remethylation, and in order to sustain S-adenosylmethionine production both in the cytoplasm and the nucleus [11, 37]. In parallel, Hcy excess can be converted by methionyl-tRNA synthetase into Hcy thiolactone which reacts with lysine residues rendering protein homocysteinylation, a PTM that has been associated with protein damage and dysfunction [76, 77]. Therefore, putative homocysteinylation of BHMT and/or the interaction targets identified in the present work may preclude their binding to BHMT and, in turn the crosstalk with other pathways. Additionally, HHcy due to liver injury or stress concurs with inflammation, a context in which transglutaminases are activated, thus promoting crosslinking and production of high-Mr complexes that may include i.e. the proinflammatory protein HMGB1 [78], in turn translocated to the cytoplasm [36]. BHMT is also a substrate of transglutaminase [33, 51], but whether this modification takes place in HHcy remains unknown. We can however speculate that in HHcy a competition for lysine residues may be established between transglutamination and homocysteinylation resulting in either high-Mr complexes or aggregates that cause protein dysfunction. Furthermore, HHcy may also result from the increased Zn^2+^ loss observed in diabetes and alcoholism, as this cation is essential for BHMT activity [25, 79]. Removal of the Zn^2+^ atom leads to an oxidized state of BHMT unable to catalyze Hcy remethylation and structural changes that seem restricted to the active site [23]. However, we cannot exclude that other subtle changes take place in the protein structure, putatively affecting protein-protein interaction surfaces such as those involved in ALDOB binding, therefore promoting/precluding the crosstalk between remethylation and glycolysis. In addition, the relevance of the BHMT-Prkaca interaction in HHcy is supported by results of alamandine treatment to revert vascular dysfunction [80]. This drug reduces HHcy, whereas inhibition of the PKA signaling pathway decreases its effectivity. Thus, it can be suggested that BHMT phosphorylation by this kinase may be needed for enzyme activation, in turn favoring an increase in Hcy remethylation.

Altogether, we have expanded the network of validated BHMT interaction targets in normal rat liver to include not only enzymes of the methionine cycle, but also of other metabolic pathways and putative regulators (Fig 11C). Crossregulatory events linking these pathways may contribute to understand the metabolic behavior observed in the large variety of pathologies where HHcy is detected.

## Supporting information

S1 Table Complete datasets obtained in AP-MS experiments. (XLSX)

S1 Appendix Original immunoblots used in the figures. (PDF)
