# Supplementary material for: Identification of hepatic protein-protein interaction targets for betaine homocysteine S-methyltransferase
Source: PLoS One. 2018 Jun 20;13(6):e0199472. doi: 10.1371/journal.pone.0199472 (PMC6010280; doi:10.1371/journal.pone.0199472)

S1 Appendix. Original immunoblots used in the figures

Fig 4. Original images of immunoprecipitations carried out for MATα1 incubated with anti-HA (left) or anti-FLAG (right)

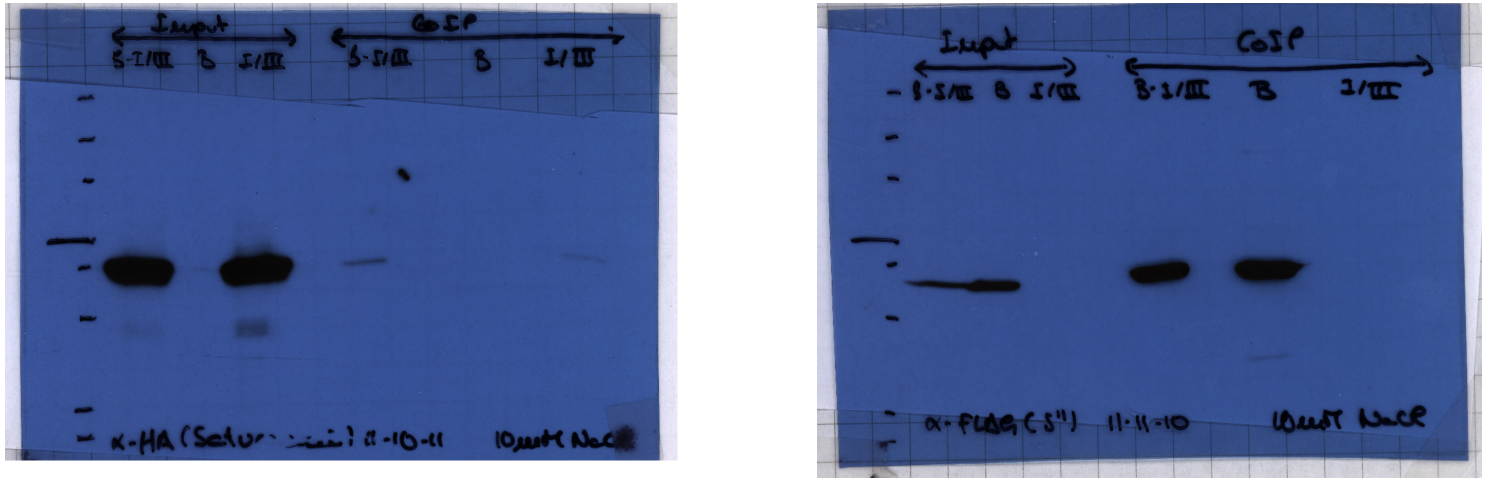

Fig 5. Original images of immunoprecipitations carried out for MATα2 incubated with anti-HA (left) or anti-FLAG (right)

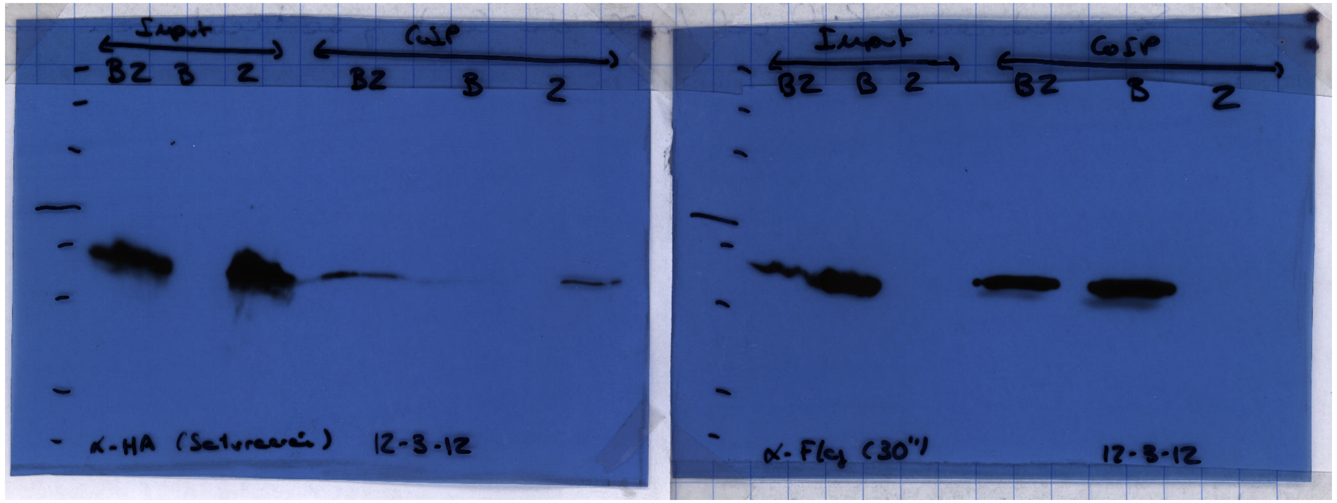

Fig 6. Original images of immunoprecipitations carried out for actin B incubated with anti-FLAG (left) or anti-HA (right)

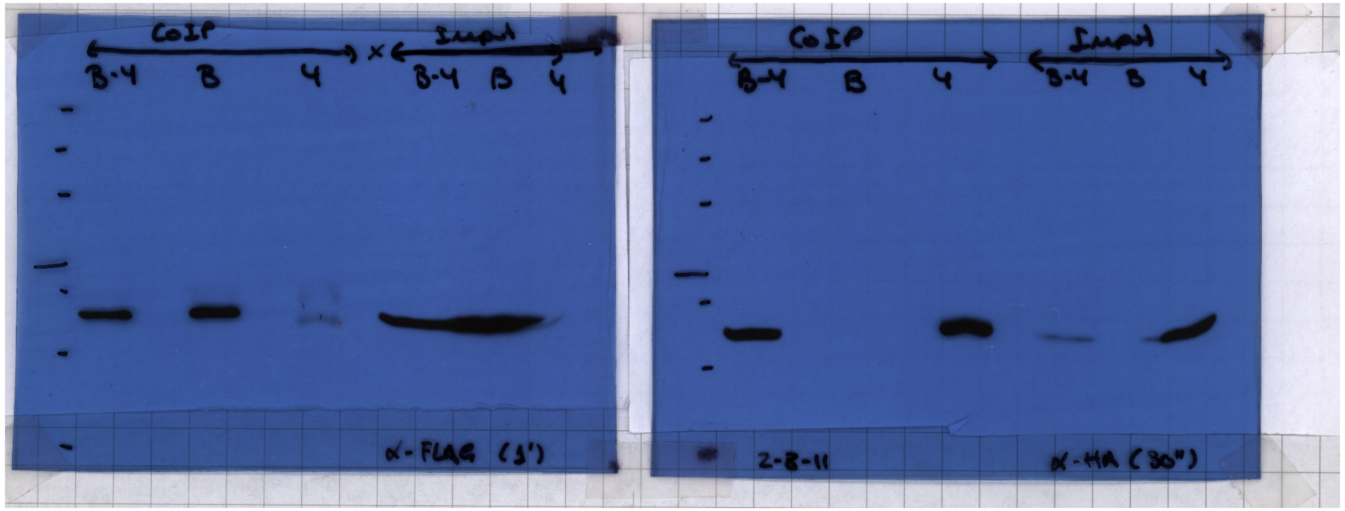

Fig 7. Original images of immunoprecipitations carried out for Ybx1 incubated with anti-HA (left) or anti-FLAG (right)

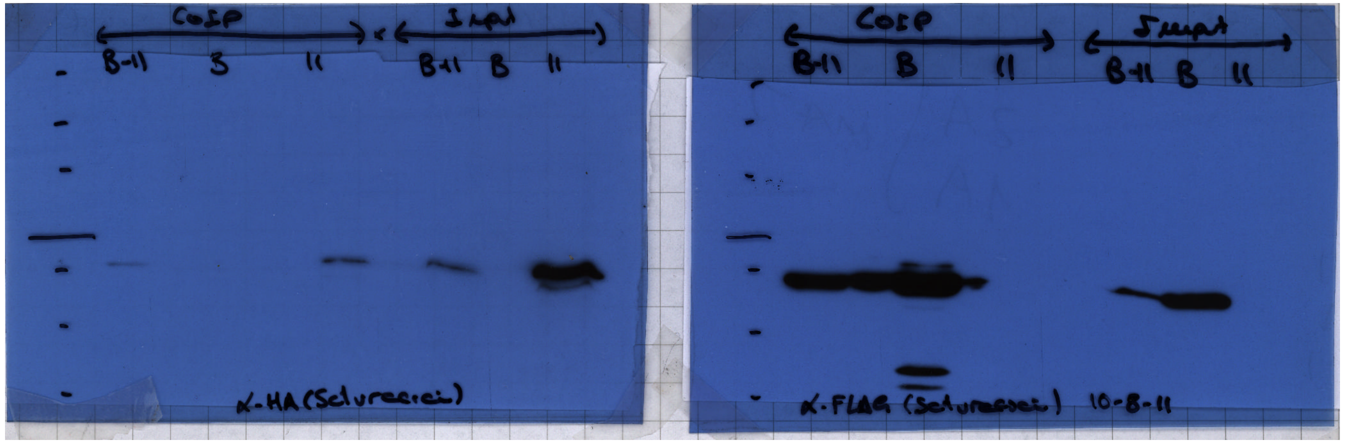

Fig 8. Original images of immunoprecipitations carried out for Hmgb1 incubated with anti-HA (left) or anti-FLAG (right)

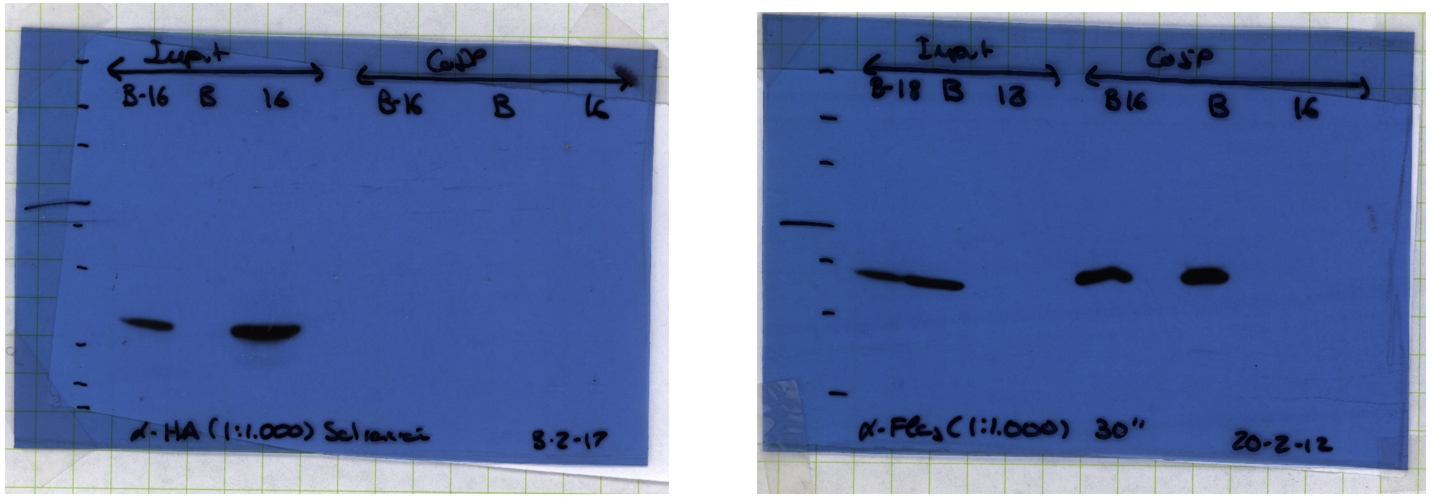

Fig 9. Original images of immunoprecipitations carried out for Prkaca incubated with anti-HA (left) or anti-FLAG (right)

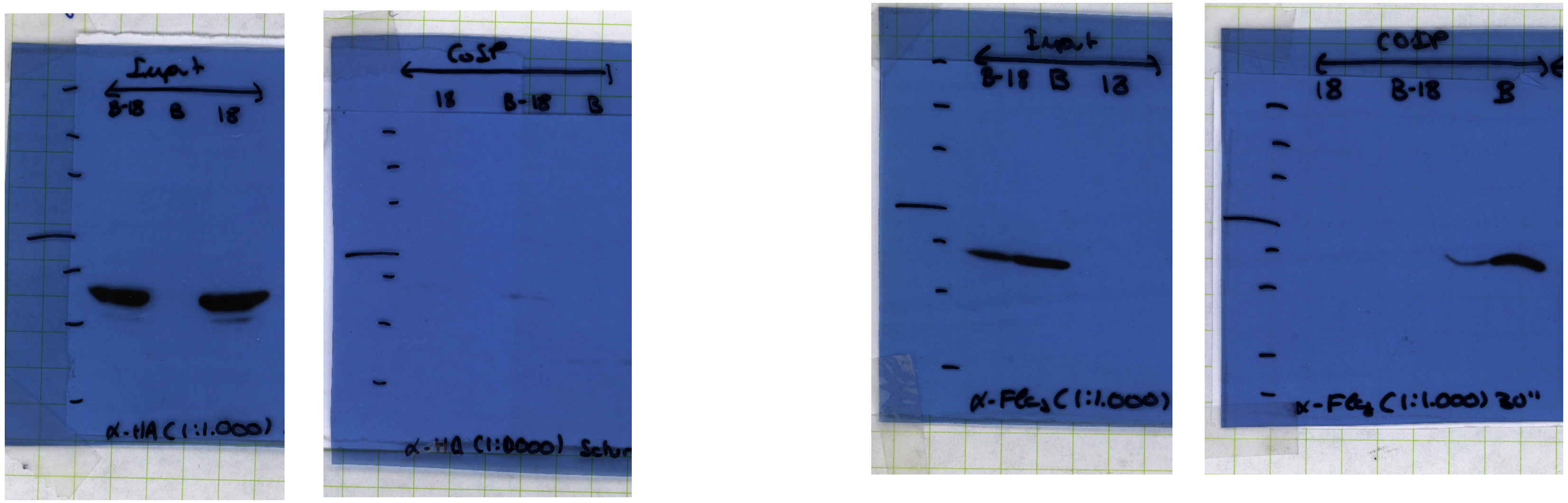

Fig 10. Original images of immunoprecipitations carried out for ALDOB (top) and HPD (down) incubated with a mixture of anti-HA and anti-BHMT

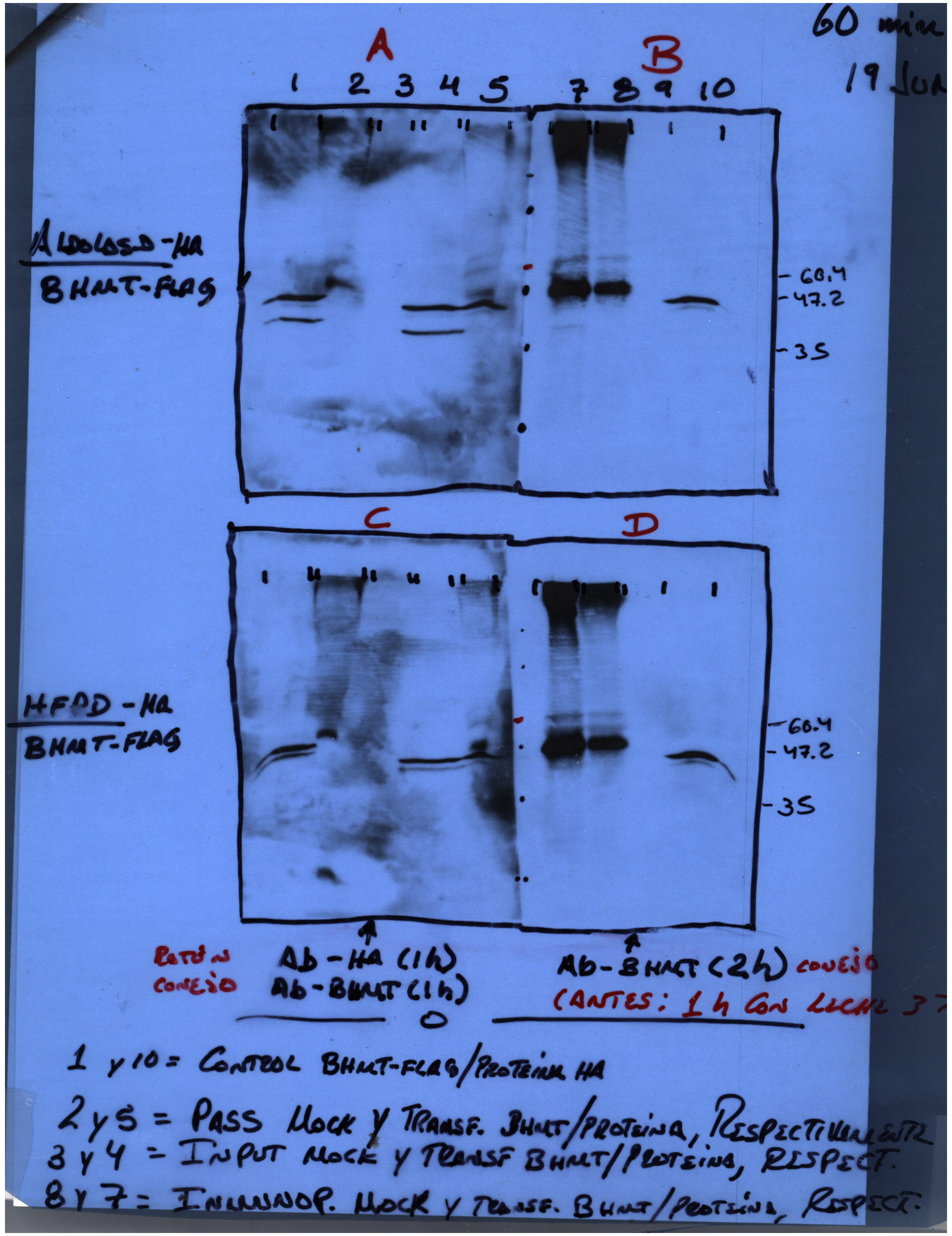

Supplement: S1 Appendix — (PDF) [file pone.0199472.s002.pdf]
